# Investigating the Chemolithoautotrophic and Formate Metabolism of Nitrospira moscoviensis by Constraint-Based Metabolic Modeling and ^13^C-Tracer Analysis

**DOI:** 10.1128/mSystems.00173-21

**Published:** 2021-08-17

**Authors:** Christopher E. Lawson, Aniela B. Mundinger, Hanna Koch, Tyler B. Jacobson, Coty A. Weathersby, Mike S. M. Jetten, Martin Pabst, Daniel Amador-Noguez, Daniel R. Noguera, Katherine McMahon, Sebastian Lücker

**Affiliations:** a Department of Civil and Environmental Engineering, University of Wisconsin—Madison, Madison, Wisconsin, USA; b Department of Microbiology, Radboud Institute for Biological and Environmental Sciences, Radboud University, Nijmegen, The Netherlands; c Department of Biotechnology, Delft University of Technology, Delft, The Netherlands; d DOE Great Lakes Bioenergy Research Center, University of Wisconsin—Madison, Madison, Wisconsin, USA; e Department of Bacteriology, University of Wisconsin—Madison, Madison, Wisconsin, USA; University of British Columbia; Oregon State University

**Keywords:** lithoautotrophic metabolism, metabolic modeling, metabolomics, proteomics, systems biology

## Abstract

Nitrite-oxidizing bacteria belonging to the genus *Nitrospira* mediate a key step in nitrification and play important roles in the biogeochemical nitrogen cycle and wastewater treatment. While these organisms have recently been shown to exhibit metabolic flexibility beyond their chemolithoautotrophic lifestyle, including the use of simple organic compounds to fuel their energy metabolism, the metabolic networks controlling their autotrophic and mixotrophic growth remain poorly understood. Here, we reconstructed a genome-scale metabolic model for Nitrospira moscoviensis (*i*Nmo686) and used flux balance analysis to evaluate the metabolic networks controlling autotrophic and formatotrophic growth on nitrite and formate, respectively. Subsequently, proteomic analysis and [^13^C]bicarbonate and [^13^C]formate tracer experiments coupled to metabolomic analysis were performed to experimentally validate model predictions. Our findings corroborate that *N. moscoviensis* uses the reductive tricarboxylic acid cycle for CO_2_ fixation, and we also show that *N. moscoviensis* can indirectly use formate as a carbon source by oxidizing it first to CO_2_ followed by reassimilation, rather than direct incorporation via the reductive glycine pathway. Our study offers the first measurements of *Nitrospira*’s *in vivo* central carbon metabolism and provides a quantitative tool that can be used for understanding and predicting their metabolic processes.

**IMPORTANCE***Nitrospira* spp. are globally abundant nitrifying bacteria in soil and aquatic ecosystems and in wastewater treatment plants, where they control the oxidation of nitrite to nitrate. Despite their critical contribution to nitrogen cycling across diverse environments, detailed understanding of their metabolic network and prediction of their function under different environmental conditions remains a major challenge. Here, we provide the first constraint-based metabolic model of Nitrospira moscoviensis representing the ubiquitous *Nitrospira* lineage II and subsequently validate this model using proteomics and ^13^C-tracers combined with intracellular metabolomic analysis. The resulting genome-scale model will serve as a knowledge base of *Nitrospira* metabolism and lays the foundation for quantitative systems biology studies of these globally important nitrite-oxidizing bacteria.

## INTRODUCTION

The oxidation of nitrite to nitrate is a key step in nitrification and the global nitrogen cycle. The process is a critical control point counteracting nitrogen loss to the atmosphere and is mediated by a phylogenetically diverse functional guild known as the nitrite-oxidizing bacteria (NOB) (1). The genus *Nitrospira* constitutes the most diverse and abundant NOB based on marker gene (16S rRNA and nitrite oxidoreductase [NXR]) and metagenomic surveys. The genus *Nitrospira* consists of at least six lineages that mediate nitrite oxidation across various habitats, including soil, freshwater, marine, terrestrial, and engineered ecosystems (1, 2). *Nitrospira* must be flexible enough to survive in the wide range of fluctuating environmental conditions characteristic of these habitats, suggesting its ecophysiology and ecological niches extend beyond those initially defined by its chemolithoautotrophic lifestyle.

Despite their recalcitrance to cultivation, recent insights driven by metagenomics have shed light on the unique features of *Nitrospira*’s carbon and energy metabolism (3). *Nitrospira* harbors novel respiratory chain enzymes for energy conservation, including an evolutionarily distinct membrane-bound periplasmic NXR and a putative cytochrome *bd*-like oxidase that may allow it to adapt to low-dissolved-oxygen environments (3). *Nitrospira* also harbors all genes for CO_2_ fixation via the reductive tricarboxylic acid cycle (rTCA) and lacks the two key genes (for ribulose bisphosphate carboxylase and phosphoribulokinase) needed to operate the Calvin-Benson-Bassham cycle (CBB) used by some NOB (3). Outside their chemolithoautotrophic growth, genomic and experimental data have revealed that *Nitrospira* spp. can use alternative substrates to fuel their carbon and energy metabolism (1, 4, 5). In addition to nitrite, some *Nitrospira* species have been experimentally shown to use formate, hydrogen, and ammonia as electron donors with oxygen or nitrate as terminal electron acceptors (4–9). For example, Nitrospira moscoviensis contains a soluble formate dehydrogenase and NiFe hydrogenase that allow growth with formate and H_2_, respectively (4, 5), whereas the recently discovered complete ammonia-oxidizing (comammox) *Nitrospira* contains pathways for both ammonia and nitrite oxidation that enable growth via complete nitrification (7, 8). In addition to their energy metabolism, fluorescence *in situ* hybridization combined with microautoradiography (FISH-MAR) experiments have also suggested that *Nitrospira* populations present within activated sludge microbial communities can assimilate pyruvate (2) and formate (10), although the carbon assimilation pathways for these substrates have yet to be determined. While this expanded metabolic versatility suggests that *Nitrospira* are adapted to dynamic environmental conditions, our ability to predict their function in natural and engineered ecosystems is constrained by the limited understanding of their metabolic network and the lack of quantitative tools to study their metabolism.

Genome-scale metabolic modeling is a powerful method for analyzing and predicting the biochemical pathways driving microbial metabolism. Such modeling approaches calculate the flow of metabolites through a reconstructed metabolic network based on relevant constraints (e.g., network stoichiometry, thermodynamics, and measured fluxes) using a technique called flux balance analysis (FBA) (11). This provides a quantitative framework for analyzing metabolism and predicting phenotypes when combined with physiological data. Moreover, genome-scale models can be used to generate testable hypotheses on the functional capabilities of organisms under defined conditions that can subsequently be tested, for instance using ^13^C isotope tracing combined with metabolomics (12).

Here, we provide the first constraint-based metabolic reconstruction and analysis of *N. moscoviensis* representing the ubiquitous *Nitrospira* lineage II. We examine the metabolism of *N. moscoviensis* growing under chemolithoautotrophic conditions and also during growth with formate as a substrate. Taking advantage of recent advances to cultivate *Nitrospira* in continuous flow membrane bioreactors (13), we subsequently validate *N. moscoviensis*’ predicted metabolic network using proteomics and ^13^C tracers combined with quantitative metabolomic analysis. Our proteomic and ^13^C metabolomic results corroborate the use of the rTCA for carbon fixation by *N. moscoviensis* during chemolithoautotrophic growth. We further show that *N. moscoviensis* does not assimilate formate directly, but instead reassimilates CO_2_ produced via formate oxidation using the rTCA cycle. The resulting genome-scale model (GEM), *i*Nmo686, will serve as a knowledge base for understanding and predicting the function of *Nitrospira* in both natural and engineered ecosystems.

(This article was submitted to an online preprint archive [14].)

## RESULTS

### Genome-scale metabolic reconstruction of Nitrospira moscoviensis.

The genome-scale metabolic network of *N. moscoviensis* (*i*Nmo686) was reconstructed from the most recent *N. moscoviensis* genome annotation (NCBI accession number NZ_CP011801), aided by reaction annotations in the MetaCyc (15) and ModelSEED databases available through the Department of Energy systems biology knowledgebase (16). Proteomic analysis of *N. moscoviensis* grown on nitrite was also performed to confirm that model reactions with nonzero flux values were detected in the proteome (see Table S1 in the supplemental material). The final reconstruction contained a total of 678 reactions, 638 metabolites, and 686 genes (see Data Set S1). Reactions for *Nitrospira*’s respiratory chain were reconstructed based on existing models of electron flow (1, 3) and assuming that the two protons produced during nitrite oxidation in the periplasm contribute to proton motive force generation. Cytoplasmic reactions for formate dehydrogenase and hydrogenase that catalyze formate oxidation to CO_2_ and hydrogen oxidation, respectively, were also included based on genomic and experimental evidence (4, 5). Formate transport and assimilation reactions mediated by a putative reductive glycine pathway (17) encoded in the genome (Data Set S1) were also included to evaluate the possibility of directly assimilating formate as a carbon source, as suggested by previous FISH-MAR observations (10).

10.1128/mSystems.00173-21.6 TABLE S1 Protein coverages of five different proteomic approaches and gene expression levels of *N. moscoviensis* CDSs during growth on nitrite. Download Table S1, XLSX file, 0.9 MB. Copyright © 2021 Lawson et al. 2021 Lawson et al. https://creativecommons.org/licenses/by/4.0/ This content is distributed under the terms of the Creative Commons Attribution 4.0 International license.

10.1128/mSystems.00173-21.9 DATA SET S1 *i*Nmo686 model reactions, metabolites, biomass equation, and FBA solutions for the model simulations performed. Download Data Set S1, XLSX file, 0.2 MB. Copyright © 2021 Lawson et al. 2021 Lawson et al. https://creativecommons.org/licenses/by/4.0/ This content is distributed under the terms of the Creative Commons Attribution 4.0 International license.

**FIG 1:**
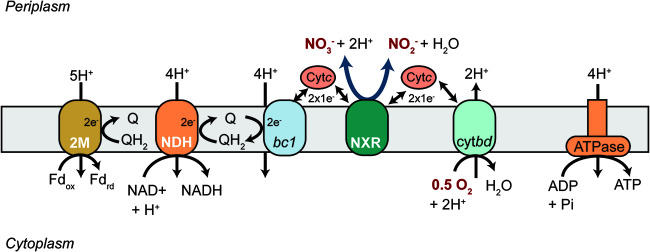
Theoretical model for the respiratory chain of Nitrospira moscoviensis as hypothesized in this study. Q, quinone; QH_2_, quinol; Fd, ferredoxin; Cyt*c*, cytochrome *c*; 2M, 2M-type complex I; NDH, NADH dehydrogenase (complex I); *bc1*, cytochrome *bc*_1_ complex (complex III); NXR, nitrite oxidoreductase; cyt*bd*, cytochrome *c* oxidase (complex IV).

Since the stoichiometry for proton translocation in *Nitrospira*’s respiratory chain complexes is unknown, values were assumed from model organisms (Data Set S1; Fig. 1). The mechanism for reducing low-potential electron carriers, such as ferredoxin, required for carbon fixation in *Nitrospira* is also unknown, but it has been hypothesized that the 2M-type NADH dehydrogenase complex (complex I) in *Nitrospira* performs this function (18). Thus, the model included this mechanism for ferredoxin reduction. All annotated biosynthetic and biodegradation reactions for amino acids, nucleic acids, carbohydrates, lipids, and cofactors were included in the reconstruction (see Data Sets 1 and 2). This encompassed all predicted central carbon metabolic reactions annotated in the genome, including reactions of the rTCA cycle, gluconeogenesis, the pentose phosphate pathway, anaplerotic reactions, one-carbon metabolism, and fatty acid metabolism. Gaps in the network were identified and filled manually to ensure that the model could grow on minimal NOB medium (see Materials and Methods).

To obtain qualitative and quantitative outputs from a genome-scale model via FBA, an objective function is required. This is typically accomplished based on a biomass objective function, which assumes that maximization of biomass growth rate is the cellular objective (19). Therefore, to generate a representative biomass objective function for *N. moscoviensis*, we experimentally determined its biomass composition (i.e., macromolecular components) during chemolithoautotrophic steady-state growth on nitrite (see Materials and Methods).

A summary of the biomass composition for *N. moscoviensis* is presented in Tables 1 and 2. These measurements, together with the genome sequence data and published fatty acid composition data (20), were used to formulate *N. moscoviensis*’ biomass objective function (Data Set S1). Growth- and nongrowth-associated maintenance energy requirements were estimated to be 535 mmol ATP g dry weight^−1^ and 0.90 mmol ATP g dry weight^−1^ h^−1^, respectively, by plotting the experimentally determined nitrite uptake rate against the growth rate and using a net ATP yield of 1.0 mmol ATP mmol NO_2_^−^-N^−1^ determined from the model (see Fig. S1).

**TABLE 1:** *N. moscoviensis* biomass composition

Molecule	% dry wt	
	Avg	SD
RNA	2.4	0.04
DNA^*a*^	1	ND^*b*^
Proteins	46.7	4.7
Carbohydrates	26.9	1.8
Lipids	16.3	3.2
Inorganics^*a*^	5	ND

a Values for DNA and inorganics were assumed based on values from Neidhardt et al. (53).

b ND, not determined.

**TABLE 2:** *N. moscoviensis* amino acid composition

Amino acid	Mass (μmol/mg dry wt)	
	Value	SD
Ala	300.6	45.7
Arg	311.8	63.9
Asp	82.3	12.3
Glu	85	16.1
Gly	825.2	116.1
His	37.7	2.6
Ile	198.7	62.1
Leu	309.2	66.6
Lys	105.9	22.2
Met	194	22.8
Phe	224.3	107.9
Pro	641	227
Ser	297.5	62.6
Thr	277.2	70
Tyr	41.9	ND^*a*^
Val	374	59.2

a ND, not determined.

10.1128/mSystems.00173-21.2 FIG S1 Maintenance energy determination based on plotting the experimentally determined nitrite uptake rate versus the growth rate. Experimental values were taken from the study by Mundinger et al. (13). Nongrowth-associated maintenance (NGAM) was determined by multiplying the *y* intercept value (0.90 mmol NO_2_^−^ g dry weight^−1^ h^−1^) by the model determined ATP yield (1 mmol ATP per 1 mmol NO_2_^−^). Growth-associated maintenance (GAM) was determined by matching the slope of the curve to that determined by the model, based on adjusting the value for GAM and setting the biomass growth rate to different values while minimizing the nitrite uptake rate. Download FIG S1, JPG file, 0.06 MB. Copyright © 2021 Lawson et al. 2021 Lawson et al. https://creativecommons.org/licenses/by/4.0/ This content is distributed under the terms of the Creative Commons Attribution 4.0 International license.

### Proteome of *N. moscoviensis* grown on nitrite.

**FIG 2:**
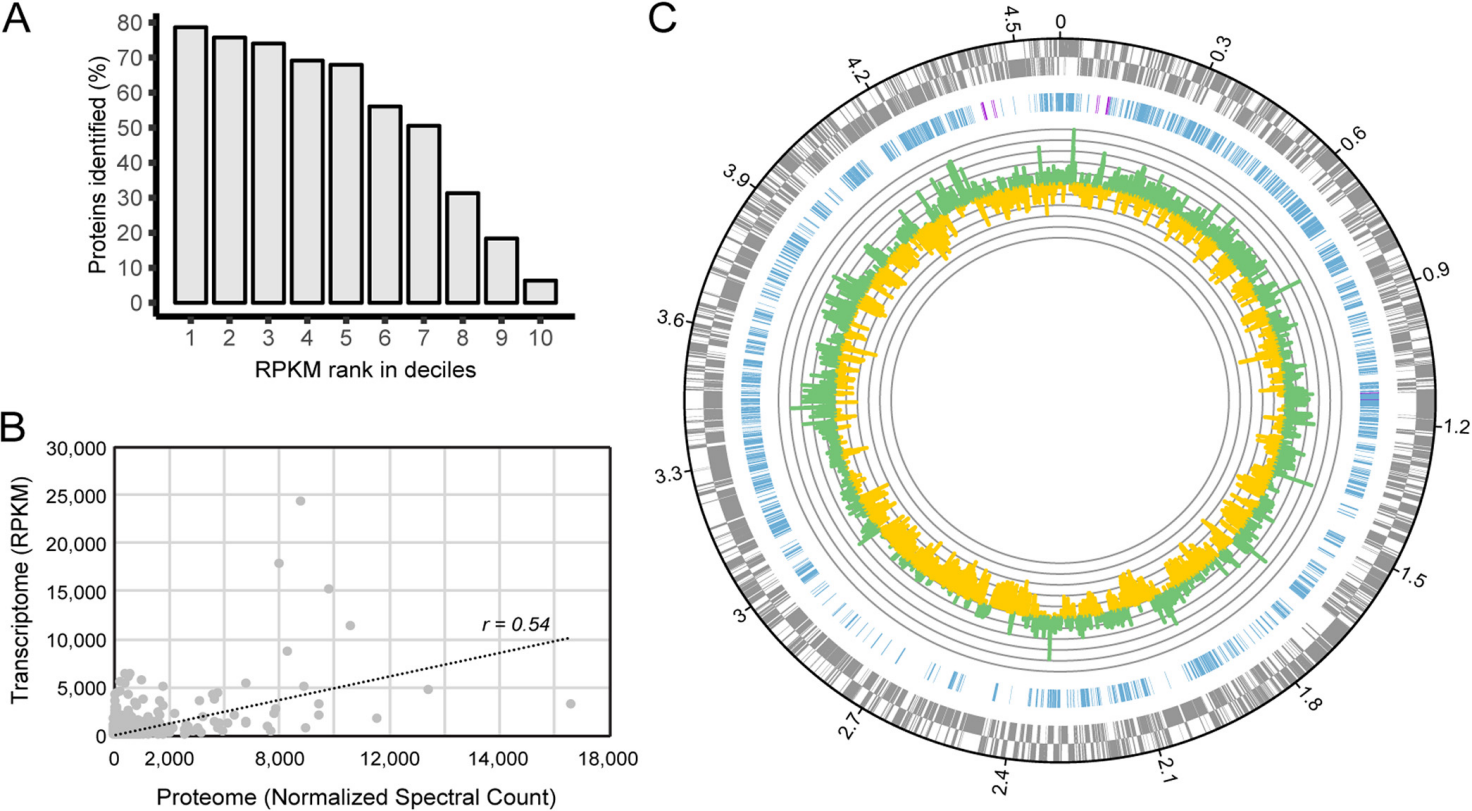
Nitrospira moscoviensis proteomic analysis. (A) Numbers of proteins (given per unique UniProt identifier) identified in the whole-cell proteome compared to their gene expression level, ranked by RPKM in deciles from high (1) to low (10). (B) Pearson correlation (*r *= 0.54, *n *= 2,788) between open reading frame (ORF) transcript abundance (RPKM) and protein abundance (normalized spectral counts) across all ORFs with detected peptides (see Table S1 in the supplemental material). (C) Genome-wide proteomic and transcriptomic profile of *N. moscoviensis* during growth on nitrite. Rings from outside to inside: (i) ORFs (± strand) predicted in the published *N. moscoviensis* genome (CP011801); (ii) CDSs corresponding to proteins identified in the whole-cell proteome, with 100% identical CDSs marked in purple; (iii) average gene expression levels (*n* = 3, with 3 technical replicates each) in log_2_ fold to median, with green representing transcription levels above and yellow representing those below the median, shown in log_2_ values.

A high-coverage proteome of *N. moscoviensis* grown on nitrite was obtained to confirm expression of the reconstructed metabolic network. Enzymatic digests using three different digestion enzymes alone and in combinations were tested to ensure a representative membrane proteome was obtained (see Text S1 for details). Whole-cell proteome analysis resulted in the detection of 2,519 of the 4,733 nonidentical proteins (53.2%) encoded in the *N. moscoviensis* genome, including 344 of the 878 nonidentical membrane proteins (39.2%) (Fig. 2; Table S1). The resulting proteome confirmed expression of genes involved in *N. moscoviensis* carbon and energy metabolism represented in the model, including the nitrite oxidation system (NXR), the reductive TCA cycle, and nitrogen assimilation pathways (see Table S2).

10.1128/mSystems.00173-21.1 TEXT S1 Proteomic method validation. Download Text S1, PDF file, 0.07 MB. Copyright © 2021 Lawson et al. 2021 Lawson et al. https://creativecommons.org/licenses/by/4.0/ This content is distributed under the terms of the Creative Commons Attribution 4.0 International license.

10.1128/mSystems.00173-21.7 TABLE S2 Protein coverages of five different proteomic approaches and gene expression levels of selected *N. moscoviensis* CDSs with function in key metabolic pathways during growth on nitrite. Download Table S2, XLSX file, 0.04 MB. Copyright © 2021 Lawson et al. 2021 Lawson et al. https://creativecommons.org/licenses/by/4.0/ This content is distributed under the terms of the Creative Commons Attribution 4.0 International license.

The measured proteome qualitatively corresponded to the recently published transcriptome of *N. moscoviensis* (13), where 86% of all detected proteins also displayed gene expression levels above the median level (Fig. 2; Table S1). However, protein and transcript abundances were only moderately correlated when quantitatively compared using normalized spectral counts as a proxy for protein abundance (*r* = 0.54) (Fig. 2B). While some of the highly transcribed open reading frames (ORFs) that lacked detection on the proteome level represent nonprotein coding sequences (such as NITMOv2_0031 and NITMOv2_0852), there were also proteins which, despite relatively high transcript levels, were not detected at all in the proteome, such as ATP-dependent RNA helicase RhlE (NITMOv2_1402) and the periplasmic phosphate-binding protein (PstS) of the phosphate ABC transporter (NITMOv2_4757). Such discrepancies can be caused by posttranscriptional regulation or limitations of the proteomic method, such as insufficient solubilization or the presence of few potential cleavage sides for the tryptic digest.

### Analysis of chemolithoautotrophic growth on nitrite.

**FIG 3:**
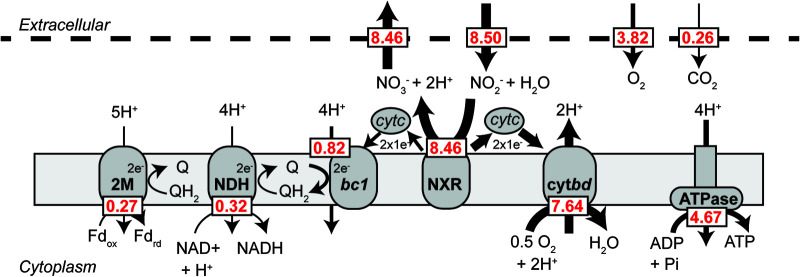
Electron flux distribution predicted via flux balance analysis during chemolithotrophic growth on nitrite. The model was constrained to a nitrite uptake rate of 8.5 mmol g dry weight^−1^ h^−1^ and the biomass growth rate was 0.006 h^−1^. Numerical values (red) are calculated fluxes in units of millimoles per gram dry weight per hour. Model reactions, compounds, and FBA solutions can be found in Data Set S1. Abbreviations are identical to those in Fig. 1.

**FIG 4:**
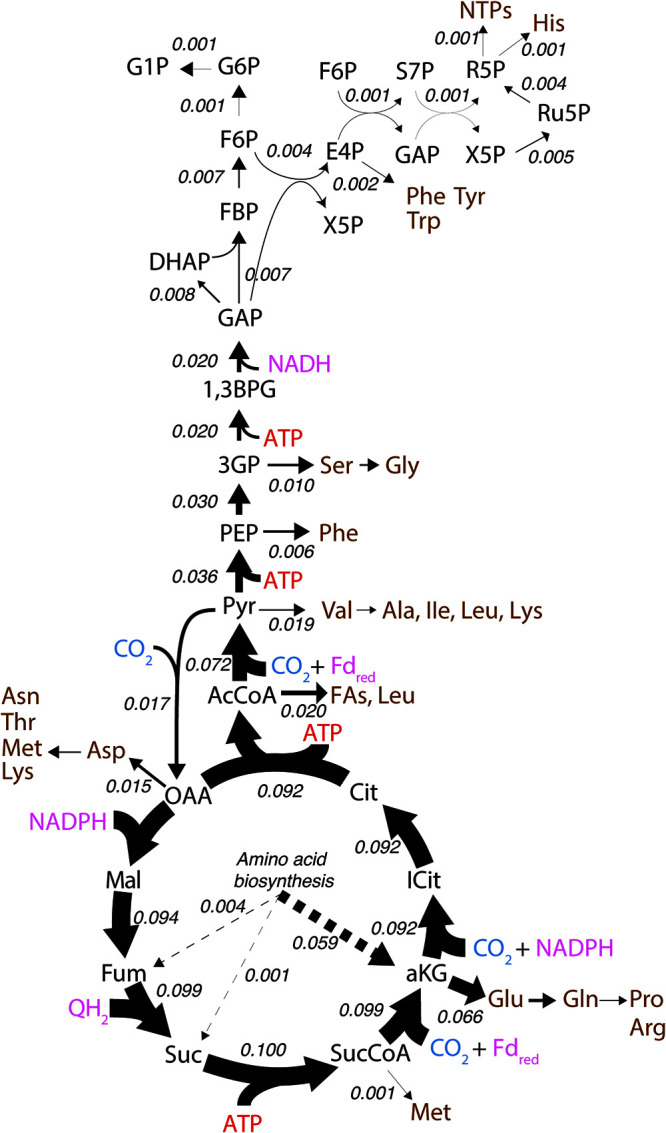
Carbon flux distribution predicted via flux balance analysis during chemolithoautotrophic growth on nitrite. The model was constrained to a nitrite uptake rate of 8.5 mmol g dry weight^−1^ h^−1^ and the biomass growth rate was 0.006 h^−1^. CO_2_ is shown in blue, amino acids and other biomass precursors are shown in brown, ATP is shown in red, and reducing equivalents are shown in pink. Numerical values are calculated fluxes in units of millimoles per gram dry weight per hour. Model reactions, compounds, and FBA solutions can be found in Data Set S1.

The *i*Nmo686 genome-scale model was first used to quantify the carbon and electron flux distribution in *N. moscoviensis* during aerobic growth on mineral medium with nitrite as an electron donor and CO_2_ as a carbon source. The model was constrained by the nitrite uptake rate measured during chemostat cultivation, and the objective function was maximizing growth. Consistent with experimental data, when the nitrite uptake rate was set to 8.5 mmol NO_2_^−^ g dry weight^−1^ h^−1^, the specific biomass growth rate equaled 0.006 h^−1^ (∼116-h doubling time) (Fig. S1). Under these conditions, approximately 66% of the oxidized NO_2_^−^ is used for growth-associated maintenance (GAM; polymerization of amino acids into proteins, RNA error checking, etc.) and 17% is oxidized for nongrowth-associated maintenance (NGAM; maintenance of chemical gradients, turgor pressure, etc.) in the form of ATP (see Fig. S2). The remaining 17% is used to generate ATP (7%) and reducing equivalents (10%) required for CO_2_ fixation and synthesis of macromolecular building blocks (e.g., central metabolites, amino acids, nucleotides, and fatty acids). Electron transport chain (ETC) reactions involved in nitrite oxidation carried the highest flux and were at an order of magnitude higher than carbon fixation fluxes, where the predicted amount of carbon fixed per mole of nitrite oxidized was 32 mol C/mol N (Fig. 3 and 4). This agrees with *N. moscoviensis*’ large maintenance energy demand and the high abundance of ETC proteins measured in the proteome (Table S2).

10.1128/mSystems.00173-21.3 FIG S2 Electron flux distribution in *N. moscoviensis.* Solid lines indicate electron flow, dashed lines indicate redox half-reactions. FBA solutions can be found in Data Set S1. Download FIG S2, PDF file, 0.4 MB. Copyright © 2021 Lawson et al. 2021 Lawson et al. https://creativecommons.org/licenses/by/4.0/ This content is distributed under the terms of the Creative Commons Attribution 4.0 International license.

The predicted carbon flux distribution in *N. moscoviensis* during chemolithoautotrophic growth (Fig. 4) showed that enzymes of the rTCA cycle, including 2-oxoglutarate:ferredoxin oxidoreductase (OFOR), succinyl coenzyme A synthetase (SCS), ATP citrate lyase, (ACL), and pyruvate:ferredoxin oxidoreductase (PFOR), carried the highest carbon flux, consistent with their primary role in CO_2_ fixation. Pyruvate carboxylase also carried high carbon flux, which is required to replenish TCA cycle intermediates used as precursors for biosynthesis (e.g., oxaloacetate). These flux values agree with the high protein expression levels of rTCA cycle enzymes measured in the *N. moscoviensis* proteome (Table S2) and gene expression recently reported in the transcriptome (13). In particular, isocitrate dehydrogenase, 2-oxoglutarate:ferredoxin oxidoreductase, and pyruvate:ferredoxin oxidoreductase were the most abundant TCA cycle enzymes (Table S2). This is consistent with the large thermodynamic barriers encountered by the consecutive reactions of alpha-ketoglutarate synthase and isocitrate dehydrogenase (Δ*G* > 40 kJ/mol), as well as pyruvate:ferredoxin oxidoreductase (Δ*G* > 14 kJ/mol) (21).

Generation of reducing equivalents for carbon fixation in *N. moscoviensis* is predicted to result from reverse electron flow from the cytochrome *c* pool (3). Overall, the model predicts that approximately 3% (0.23 mmol g dry weight^−1^ h^−1^) of the oxidized nitrite is used to generate quinol (largely for nitrite assimilation via octaheme nitrite reductase and reduction of fumarate via succinate dehydrogenase), 4% (0.32 mmol g dry weight^−1^ h^−1^) is used to generate NADH (via complex I), and 3% (0.27 mmol g dry weight^−1^ h^−1^) is used to generate reduced ferredoxin (via the 2M-type complex I) (Fig. S2). Reduction of NADP^+^ to NADPH, the main electron carrier for biosynthetic reactions, was predicted to occur through NAD(P)^+^ transhydrogenase. While *N. moscoviensis* harobrs three NAD(P)^+^ transhydrogenases, only NITMOv2_1092 was detected in the proteome (Table S1), indicating that this is the main transhydrogenase during chemolithoautotrophic growth.

### Confirmation of autotrophic metabolism with [^13^C]bicarbonate tracer experiments.

To confirm the biosynthetic pathways predicted by genome-scale modeling, isotopic tracers combined with high-resolution metabolomics was used to follow ^13^C-labeled bicarbonate incorporation into the metabolome of *N. moscoviensis.* Cells were grown in a membrane bioreactor under steady-state conditions, and [^13^C]bicarbonate was rapidly introduced into the bioreactor, which equilibrated with the dissolved inorganic carbon (DIC) pool in the liquid medium to approximately 65% ^13^C enrichment. Following [^13^C]bicarbonate addition, multiple metabolite samples were collected from the bioreactor over a 2-h time period by rapid quenching and extraction of *N. moscoviensis* cells. A time zero sample corresponding to the period immediately before [^13^C]bicarbonate addition was also collected as a control.

**FIG 5:**
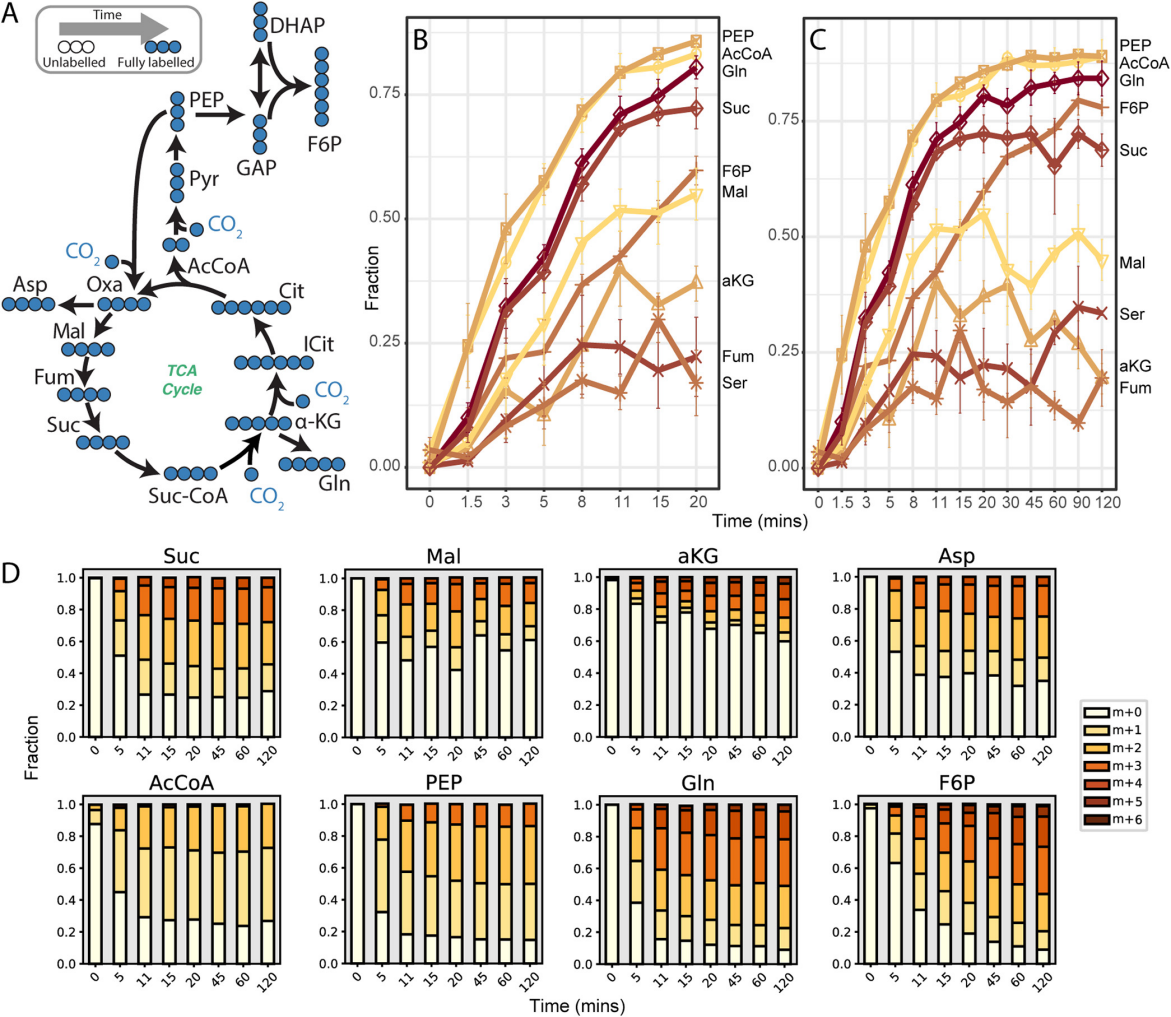
Dynamic ^13^C labeling of selected central carbon metabolites during [^13^C]bicarbonate tracer experiments. (A) Expected ^13^C labeling of central metabolism over time. ^13^C enrichment of selected metabolites over 20 (B) and 120 min (C). ^13^C enrichment values were normalized to a tracer ^13^C fraction of 1. (D) Mass isotopomer distributions for selected metabolites. AcCoA, acetyl-CoA, aKG, alpha-ketoglutarate, Asp, aspartic acid, F6P, fructose 6-phosphate, Fum, fumarate, Gln, glutamine, Mal, malate, PEP, phosphoenolpyruvate, Ser, serine, Suc, succinate. All measured metabolite mass isotopomer distributions can be found in Data Set S2.

Consistent with the operation of the rTCA cycle, high ^13^C label incorporation was measured in phosphoenolpyruvate (PEP), acetyl coenzyme A (acetyl-CoA), succinate, and aspartic acid (used as oxaloacetate surrogate) (Fig. 5). Other TCA cycle metabolites showed considerable ^13^C enrichment, including malate, fumarate, and alpha-ketoglutarate, although to a lower extent than for PEP, acetyl-CoA, succinate, and aspartic acid (Fig. 5). This suggested that the ^13^C enrichment of malate, fumarate, and alpha-ketoglutarate may have been diluted by inactive pools of these metabolites that were not labeled. Further evidence for this was observed from the faster labeling of glutamate and glutamine than that of alpha-ketoglutarate, which was unexpected because these amino acids are derived from alpha-ketoglutarate (Fig. 5; Data Set S2). We hypothesize that these patterns may reflect substrate channeling in the rTCA cycle of *N. moscoviensis*, which has previously been observed in the TCA cycle of other organisms (22–24).

10.1128/mSystems.00173-21.10 DATA SET S2 Metabolite mass isotopomer distributions and ^13^C enrichment values from the [^13^C]bicarbonate and [^13^C]formate isotope tracer experiments. Download Data Set S2, XLSX file, 0.07 MB. Copyright © 2021 Lawson et al. 2021 Lawson et al. https://creativecommons.org/licenses/by/4.0/ This content is distributed under the terms of the Creative Commons Attribution 4.0 International license.

In addition to the TCA cycle, fast labeling of 3-phosphoglycerate, fructose 6-phosphate, glucose 6-phosphate, and sedoheptulose 7-phosphate was observed (Fig. 5; Data Set S2). This is consistent with the use of gluconeogenesis and the pentose phosphate pathway for synthesis of precursor metabolites in *N. moscoviensis.*

### Potential for formatotrophic and mixotrophic growth.

It has been demonstrated that several *Nitrospira* species can use formate as an energy source and that their genomes contain a formate transporter (FocA) and a soluble NAD^+^-reducing formate dehydrogenase (FDH), which catalyzes the oxidation of formate to CO_2_ with concomitant reduction of NAD^+^ to NADH (5, 9). Notably, *N. moscoviensis* and the marine Nitrospira marina were reported to grow less efficiently on formate than on nitrite (5, 9), a trend we confirmed for *N. moscoviensis* in batch experiments (see Fig. S4A). Furthermore, growth on formate was tested over a range of pH conditions, as previous studies indicated that formate oxidation may have a different pH optimum than nitrite oxidation (25, 26). However, growth with formate as the sole energy source was even less efficient at lower pH values (Fig. S4B), indicating that the low growth efficiency on formate at pH 7.7 was not due to a difference in pH optima for formate oxidation.

10.1128/mSystems.00173-21.4 FIG S3 Venn diagrams depict the numbers of proteins identified (with number of unique peptides of ≥2) unique and common in different proteome preparations. (A) Comparison of the whole-cell and the membrane proteomes. A protein was counted as identified in the membrane proteome when it was identified with ≥2 unique peptides in at least one of the five membrane proteome preparations. Given in parentheses are the ratios of membrane proteins (containing ≥1 TMH) to soluble proteins. (B) Comparison of five membrane proteome preparations using different enzymatic digests. Given in parentheses are the total numbers of proteins identified in the sample. (C) Relative proteome coverages of the whole-cell proteome and five different membrane proteome preparations given per number of transmembrane helices. The relative coverage is given as a percentage of the total number of nonidentical proteins predicted to contain 0 to 18 transmembrane helices. Given in parentheses are the total numbers of proteins containing 0 to 18 transmembrane helices. Download FIG S3, PDF file, 0.9 MB. Copyright © 2021 Lawson et al. 2021 Lawson et al. https://creativecommons.org/licenses/by/4.0/ This content is distributed under the terms of the Creative Commons Attribution 4.0 International license.

10.1128/mSystems.00173-21.5 FIG S4 (A) Biomass growth rates on formate and nitrite calculated from the study by Koch et al. (5) and the current study (pH 7.7). The corresponding substrate uptake rates reported by Koch et al. (5) were approximately as follows: nitrite, 8.7 mmol g dry weight^−1^ h^−1^; and formate, 6.0 mmol g dry weight^−1^ h^−1^. (B) Growth of *N. moscoviensis* using formate (yellow) or nitrite (red) as electron donor with oxygen as the terminal electron acceptor at different pHs. *, *P* ≤ 0.1; **, *P* ≤ 0.05. Download FIG S4, PDF file, 0.3 MB. Copyright © 2021 Lawson et al. 2021 Lawson et al. https://creativecommons.org/licenses/by/4.0/ This content is distributed under the terms of the Creative Commons Attribution 4.0 International license.

**FIG 6:**
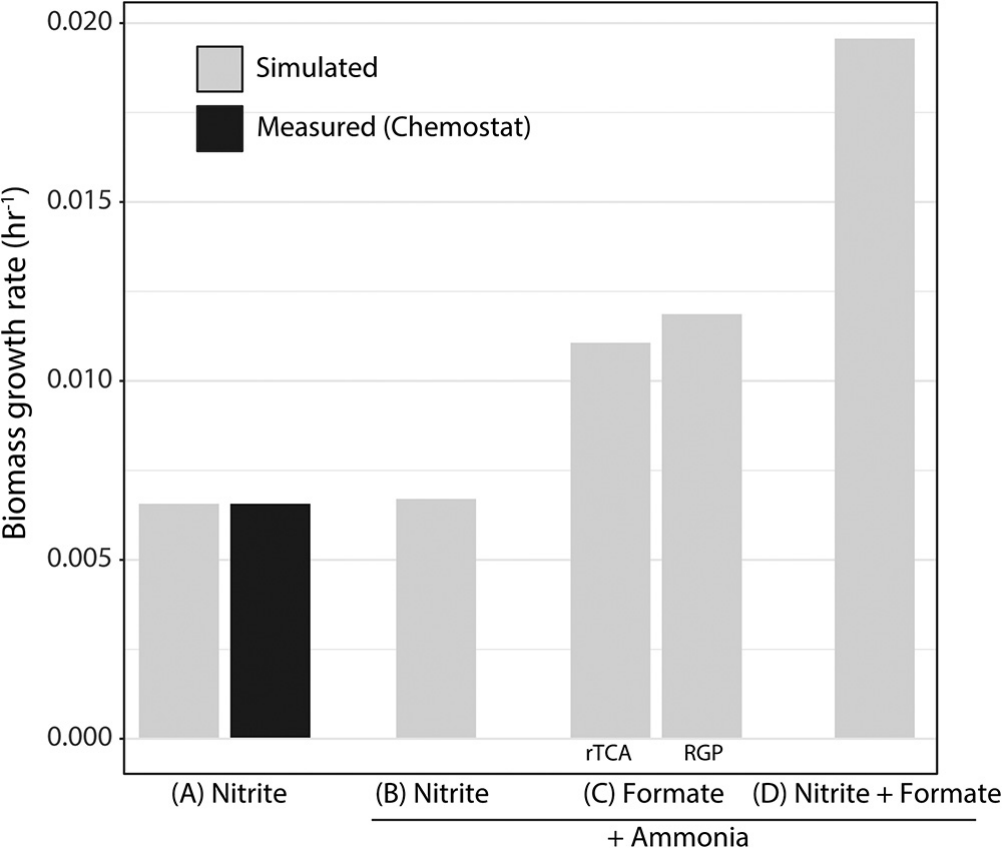
Model predictions for *N. moscoviensis* growth rate utilizing different substrates (nitrite, formate, and formate plus nitrite) and different formate assimilation pathways. rTCA, reductive TCA cycle; RGP, reductive glycine pathway. The substrate uptake fluxes in each scenario were as follows: nitrite, 8.5 mmol g dry weight^−1^ h^−1^; formate, 0 mmol g dry weight^−1^ h^−1^ (A); nitrite, 8.7 mmol g dry weight^−1^ h^−1^; formate, 0 mmol g dry weight^−1^ h^−1^ (B); nitrite, 0 mmol g dry weight^−1^ h^−1^; formate, 6.0 mmol g dry weight^−1^ h^−1^ (C); nitrite, 8.7 mmol g dry weight^−1^ h^−1^; formate, 6.0 mmol g dry weight^−1^ h^−1^ (D). Scenario A substrate uptake fluxes were based on measured values from chemostat experiments conducted in this study; scenarios B to D substrate uptake fluxes were based on measured values from batch experiments performed by Koch et al. (5) (provided in Fig. S4). In scenario C, the RGP was inactivated by setting the flux through the formate-tetrahydrofolate ligase reaction (rxn00690_c0) to zero. Model reactions, compounds, and FBA solutions can be found in Data Set 1.

Additionally, we used the model to explore predicted growth efficiency and metabolism of *N. moscoviensis* on formate compared to those under chemolithoautotrophic conditions. Comparisons were performed on minimal NOB medium plus ammonia with the following substrates: (i) nitrite and O_2_, (ii) formate and O_2_, and (iii) nitrite plus formate and O_2_. The nitrite uptake rate was derived from chemostat measurements, whereas the formate uptake rate was estimated from data provided by Koch et al. (5) (Fig. S4A). In contrast to the reported slower growth on formate, the model predicted that formate or formate plus nitrite utilization would increase the growth rate of *N. moscoviensis* by approximately 1.9- or 3.0-fold, respectively (Fig. 6). Here, electrons derived from formate oxidation were predicted to drive energy conservation via the electron transport chain while also providing reducing equivalents for biosynthesis. A possible mechanism to explain the slower growth of *N. moscoviensis* on formate could be that complex I is constrained or limited in the oxidative direction, as this enzyme normally functions reductively to generate NADH via reverse electron flow in *Nitrospira* (3). Under such a scenario, a highly active FDH would generate reduction equivalents from formate oxidation (i.e., NADH) faster than their oxidation by the electron transport chain, resulting in an electron imbalance that would have to be alleviated by, for example, the secretion of a reduced product. We used the model to test this hypothesis by fixing the formate oxidation rate to the measured value and constraining flux through complex I to the measured growth rate (based on rates by Koch et al. [5], provided in Fig. S4A). Indeed, this resulted in model solutions that could balance electrons via several different central carbon reactions of the TCA cycle and/or amino acid biosynthesis followed by product secretion (Data Set S1, “FBA solutions”). However, this hypothesis remains untested and requires experimental investigation.

**FIG 7:**
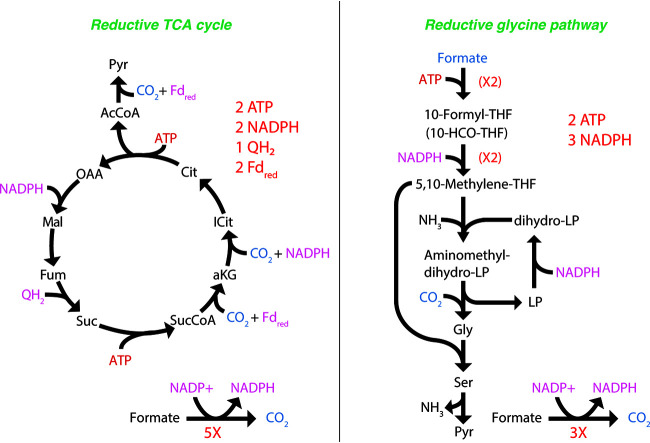
Comparison of the reductive TCA cycle and the reductive glycine pathway.

Besides its function as an energy source, formate has also been shown to be used as a carbon source by uncultured *Nitrospira* species (10), although the pathway for assimilation currently remains unclear. Metabolic reconstruction suggested that formate could potentially be assimilated either indirectly through oxidation to CO_2_ and subsequent assimilation via the rTCA cycle or directly via the reductive glycine pathway (27), for which all proteins are encoded in the genome (Data Set S1; Table S2). We thus examined the growth benefits of using either the rTCA cycle or the reductive glycine pathway for formate assimilation by turning on/off key reactions in the model. FBA predicted that *N. moscoviensis* growth rates would improve approximately 4% by directly assimilating formate via the reductive glycine pathway versus that indirectly via the rTCA cycle (Fig. 6). This is because the reductive glycine pathway consumes less energy than the rTCA cycle, as low-potential electron carriers (i.e., reduced ferredoxin) are not required, and in total, four electrons less are required per pyruvate molecule formed from formate instead of CO_2_ (Fig. 7). In this scenario, approximately 6% of the formate would be directly assimilated via the reductive glycine pathway rather than oxidizing all formate to CO_2_ followed by reassimilation (Data Set S1 “FBA solutions”).

### [^13^C]formate tracer experiments suggest formate is indirectly assimilated via CO_2_ fixation.

**FIG 8:**
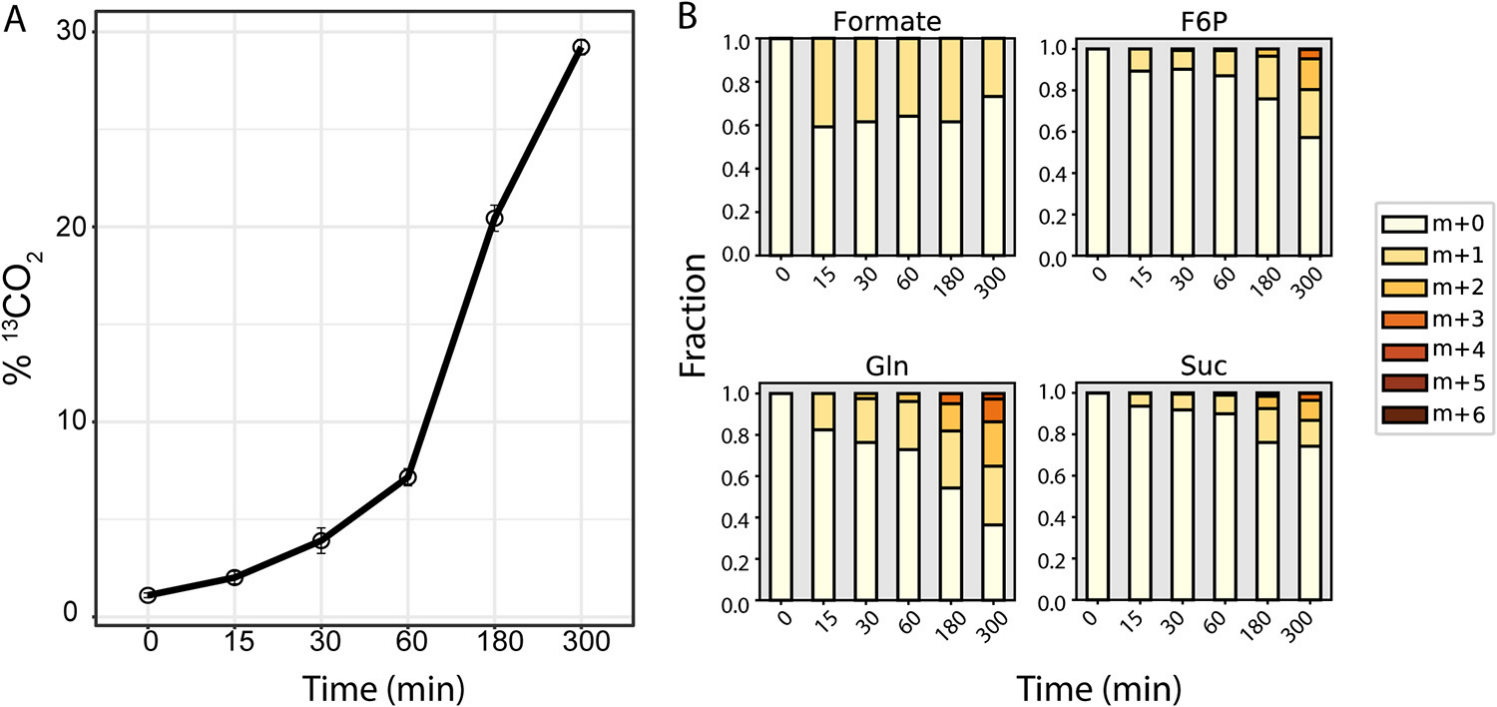
Oxidation and assimilation of [^13^C]formate by *N. moscoviensis.* (A) Production of ^13^CO_2_ from [^13^C]formate oxidation. (B) Mass isotopomer distributions for selected metabolites during batch [^13^C]formate tracer experiments. All measured metabolite mass isotopomer distributions can be found in Data Set S2.

To experimentally determine the assimilation pathway of formate in *N. moscoviensis*, isotopic tracer experiments with [^13^C]formate were performed with batch cultures in sealed serum bottles. Cultures were first acclimated to growth on 0.5 mM unlabeled formate without the presence of nitrite for 24 h. Subsequently, 1 mM [^13^C]formate was added to the cultures, and intracellular metabolome and gas headspace samples were collected before addition of [^13^C]formate and after 15, 30, 60, 180, and 300 min for isotopic analysis. Consistent with [^13^C]formate oxidation to CO_2_, continuous production of ^13^CO_2_ in the headspace gas was observed during the experiment (Fig. 8A). Intracellular formate was measured to be ∼40% ^13^C enriched from 15 min onwards, consistent with formate transport into the cell (Fig. 8B). However, the majority of measured metabolites had no detectable ^13^C enrichment, expect for succinate, glutamate, glutamine, and fructose 6-phosphate (Data Set S2; Fig. 8B). Moreover, mass isotopomer distributions for these metabolites showed a consistent increase in labeled carbons overtime (Fig. 8B), similar to results from [^13^C]bicarbonate experiments (Fig. 3). Together, these results suggest that formate was being oxidized to CO_2_ and then assimilated via the rTCA cycle and that the reductive glycine pathway is not active in *N. moscoviensis*.

## DISCUSSION

Previous studies have shown the utility of constraint-based reconstruction and analysis for exploring the metabolic capabilities of microorganisms that have important environmental roles (28–30). The model (*i*Nmo686) provides a framework for examining *Nitrospira* metabolism at a systems level and will serve as a knowledge base that can be continually refined to drive understanding and improve predictions of *Nitrospira* ecophysiology. Our analysis shows that the model quantitatively predicts *N. moscoviensis* chemolithoautotrophic growth on nitrite. Flux balance analysis provided estimates for the amount of nitrite used for energy conservation and CO_2_ fixation into biomass, offering a quantitative model for linking catabolism and anabolism. The model also accurately provides quantitative estimates of carbon and electron flux distribution, reinforcing the metabolic network predicted based on the proteomic measurements from this study and from previous genomic and transcriptomic analyses of *Nitrospira* (3, 13).

^13^C labeling metabolomic analysis revealed a complex picture for central carbon metabolism in *Nitrospira*. While our results validated that *Nitrospira* uses the rTCA cycle for CO_2_ fixation during chemolithoautotrophic growth, ^13^C enrichment values revealed extensive unlabeled pools of central carbon intermediates in the metabolome (Fig. 5). Because all ^13^C enrichment values increased monotonically, we reasoned that turnover of carbon storage reserves or macromolecules was not responsible for this observation. However, compartmentalization of metabolism or substrate channeling may offer a possible explanation. In cells that contain multiple pools of the same metabolite (e.g., cells with mitochondria), differences in their labeling will result in an aggregated measurement of the mixed pool because they are extracted together. If only one of those pools is actively participating in metabolism, the aggregation of both labeled and unlabeled pools will serve to dilute the overall ^13^C enrichment. While electron micrograph images suggest that no intracytoplasmic membranes or carboxysomes are present in *N. moscoviensis* cells (6, 31, 32), it is possible that separate metabolite pools may arise from certain enzymes/pathways having different subcellular locations within the cytoplasm (33). However, this requires further investigation.

An alternative and maybe more likely mechanism to explain the low ^13^C enrichment levels of some metabolites is substrate channeling, the direct passing of pathway intermediates between enzyme active sites without escaping into the cytoplasm, facilitated by noncovalent dynamic enzyme complexes (34, 35). This can result in lower-than-expected ^13^C enrichment values due to “leaked” metabolites that create a separate cytoplasmic pool with a much slower turnover rate (34, 35). Given that several metabolites immediately downstream of the rTCA cycle (aspartate, glutamate, and glutamine) were more strongly labeled than their precursor TCA intermediates, we suspect that rTCA cycle enzymes may interact to form a supramolecular complex, or metabolon, to efficiently transport reactants between enzyme active sites. This brings the advantage that high local substrate concentrations enable higher pathway fluxes, and intermediates can be protected from the bulk phase, limiting competition between competing pathways and protecting the cell from toxicity (34, 36). Given the significant thermodynamic barriers encountered in the rTCA by the consecutive operation of alpha-ketoglutarate synthase and isocitrate dehydrogenase (Δ*G* > 40 kJ/mol) as well as pyruvate synthase (Δ*G* > 14 kJ/mol) (21), substrate channeling maybe be important for overcoming these unfavorable reactions in addition to their indirect coupling to ATP hydrolysis via succinyl-CoA synthetase and ATP citrate lyase. Further confirmation of this channeling could be obtained through *in vivo* cross-linking coupled to proteomic analysis, as has been done to investigate the malate dehydrogenase-citrate synthase-aconitase complex of the oxidative TCA cycle in other organisms (23, 24).

In addition to chemolithoautotrophic growth on nitrite, genome-scale modeling allowed us to assess different hypotheses regarding formatotrophic and mixotrophic growth of *N. moscoviensis*. *Nitrospira* species have been observed to oxidize formate to CO_2_ for energy conservation under laboratory conditions (5) and also to assimilate formate *in situ* during wastewater treatment (10). However, while our analysis predicted that *N. moscoviensis* should grow 2 to 3 times faster on formate than on nitrite, batch experiments confirmed the previously reported reduced growth efficiency of *N. moscoviensis* on formate (5).

Interestingly, *N. marina* also showed poor growth with formate compared to that with nitrite (9), even though it possesses a cytoplasmic formate dehydrogenase that is only distantly related to the one of *N. moscoviensis*. Therefore, it is tempting to speculate that poor growth on formate might be a general feature of *Nitrospira* caused by its overall metabolism. Our modeling analysis suggested that one possible mechanism to explain this might be that the rate of formate oxidation exceeds the rate that electrons can flow through the electron transport chain, resulting in NADH accumulation that requires electrons to be segregated via secreted carbon metabolites, diverting carbon from cell growth. A possible alternative explanation for inefficient growth on formate might be an increased production of reactive oxygen species (ROS) by a respiratory chain that is potentially not well adapted for electron flow in the oxidative direction. The metabolic cost for increased ROS defense might result in low growth efficiency. However, the exact metabolic mechanism resulting in poor growth on formate awaits further physiological analysis. In addition, it also remains to be tested whether all *Nitrospira* species grow slower using formate as an electron donor and whether other environmental conditions can boost formatotrophic growth.

Genome-scale modeling further allowed us to generate hypotheses on the use of formate as a carbon source for *N. moscoviensis* anabolism. While modeling predicted that small growth improvements would be achieved by directly assimilating formate via the reductive glycine pathway, *in vivo* [^13^C]formate tracer experiments demonstrated that *N. moscoviensis* adheres to its autotrophic lifestyle, assimilating CO_2_ derived from formate oxidation via the rTCA cycle. This may be an evolutionary adaptation to avoid energetic costs associated with remodeling its proteome in environments where formate may only become transiently available, for example, at oxic-anoxic interfaces common to the habitats of *Nitrospira* (1). Together with the poor growth on formate, this observation highlights the importance of validating model predictions with experimental measurements.

In conclusion, our work provides the first genome-scale reconstruction and analysis of *Nitrospira* metabolism, offering unique insights on its versatile ecophysiology. The genome-scale model (*i*Nmo686) provides quantitative estimates of chemolithoautotrophic growth and pathway fluxes and serves as a valuable tool for hypothesis-driven discovery. Our ^13^C labeling metabolomic results also provide the first insights on *Nitrospira*’s *in vivo* central carbon metabolism, confirming the high activity of the rTCA cycle for CO_2_ fixation. Future efforts to combine ^13^C metabolomics with genome-scale modeling should provide a valuable approach for quantitatively understanding the regulation of *Nitrospira* metabolism under different environmental conditions and microbe-microbe interactions. This will further expand the systems biology framework developed in this study, ultimately leading to the systematic prediction and control of *Nitrospira* metabolism in natural and engineered ecosystems.

## MATERIALS AND METHODS

### Cultivation of *N. moscoviensis* cells.

*N. moscoviensis* M-1 was grown in NOB mineral salts medium for lithoautotrophic growth as described in Spieck and Lipski (37) except that CaCO_3_ was replaced with CaCl_2_·2H_2_O at the same concentration, and the following trace element composition was used per liter of medium: 34.4 μg of MnSO_4_·1H_2_O, 50 μg of H_3_BO_3_, 70 μg of ZnCl_2_, 72.6 μg of Na_2_MoO_4_·2H_2_O, 20 μg of CuCl_2_·2H_2_O, 24 μg of NiCl_2_·6H_2_O, 80 μg of CoCl_2_·6H_2_O, and 2,000 μg of FeSO_4_·7H_2_O. Nitrilotriacetic acid was added equimolar to all trace elements as a complexing agent.

*N. moscoviensis* was cultivated in a 7-liter membrane bioreactor (MBR) inoculated with an active batch culture and operated as described by Mundinger et al. (13). The working volume of the reactor was 3 liters and included pH, dissolved oxygen, temperature, and level controls (all by Applikon Biotechnology B.V., Schiedam, The Netherlands). The bioreactor was continuously sparged with Ar/CO_2_ (95%:5% [vol/vol]) and air at a rate of 10 ml/min to maintain a dissolved oxygen concentration of ∼30%. pH was controlled at 7.7 using a 1 M KHCO_3_ buffer. Temperature was maintained at 39°C using a loop-type heat exchanger, and the reactor was continuously stirred at 150 rpm. Reactor and all cultivation media and solutions were sterilized by autoclaving or sterile filtration prior to use, and the reactor was operated aseptically to maintain culture purity. Nitrite and nitrate concentrations were check daily to ensure all nitrite was consumed stoichiometrically to nitrate (Nitrite test strips MQuant; Merck, Darmstadt, Germany) and that nitrite was always limiting. Cultures were maintained in steady-state growth at a dilution rate of 0.006 h^−1^ and a substrate feeding rate of 2 to 2.5 mmol NO_2_^−^ liter^−1^ day^−1^.

### Genome-scale model reconstruction and analysis.

The genome-scale metabolic model of *N. moscoviensis* (*i*Nmo686) was reconstructed from the NCBI’s genome sequence for *N. moscoviensis* (accession number NZ_CP011801.1) using the ModelSEED pipeline (38) implemented in KBase (16), followed by manual curation using the MetaCyc database (15) and available literature (1, 3, 5). The model was gap filled manually through the addition of reactions not annotated in the genome to ensure that all biomass components could be produced on NOB minimal medium. Growth- and nongrowth-associated maintenance energy requirements were determined by plotting experimentally measured nitrite uptake rates as a function of the growth rate set during steady-state bioreactor cultivation (39). The biomass equation was derived from biomass composition measurements of *N. moscoviensis* (see Data Set S1 in the supplemental material). The model was formulated in Systems Biology Markup Language (SBML) level 3 version 1.0 and is available at GitHub (https://github.com/celawson87/Nitrospira-moscoviensis-GEM). Flux balance analysis was used to simulate *in silico* growth by solving the linear program:

$$\text{Max}\;v_{\text{biomass}},$$

s.t.,

S×v=0,

$$v_{\text{min}}\;\leq \;v\;\leq \;v_{\text{max}},$$

where *v*_biomass_ is the flux through the biomass objective function, *S* is the stoichiometric matrix generated from the reconstruction with rows representing metabolites, columns representing reactions, and entries representing metabolite stoichiometric coefficients, *v* is the vector of steady-state reaction fluxes, and *v*_min_ and *v*_max_ are the minimum and maximum allowable reaction fluxes, respectively. Flux balance analysis was performed in Python version 3.7.2 using the COBRApy package (40).

### Biomass composition analysis.

Cultures were centrifuged (10,000 rpm, 15 min, 4°C) to obtain cell pellets, which were subsequently freeze-dried prior to analysis. Total protein concentration was determined using the Pierce bicinchoninic acid (BCA) protein assay kit (Thermo Fisher Scientific), and amino acid composition was determined according to Carnicer et al. (41) using a Varian 920-LC high-performance liquid chromatography amino acid analyzer. Total carbohydrates were determined using the phenol-sulfuric acid method (42). Total lipid content was determined via the sulfo-phospho-vanillin reaction (43), and the lipid composition for *N. moscoviensis* was that reported by Lipski et al. (20). Total RNA and DNA content was determined according to Benthin et al. (44). Total inorganic content was determined by combustion of freeze-dried biomass in an oven at 550°C for 12 h. Lipid headgroup composition, ion composition, and soluble pool composition were derived from the Escherichia coli biomass equation reported for iAF 1260 (45).

### ^13^C isotopic tracer experiments.

^13^C-labeled sodium bicarbonate was rapidly introduced (within 1 min) into the bioreactor containing *N. moscoviensis* cells growing under steady-state conditions to a final concentration of approximately 30 mM. Following ^13^C-label introduction, samples were rapidly withdrawn from the reactor at time points 0, 1.5, 3, 5, 8, 11, 15, 20, 30, 45, 60, 90, and 120 min. Samples were immediately filtered (Millipore 0.45-μm hydrophilic nylon filter HNWPO4700) using a vacuum pump to remove extracellular medium, and filters were placed face down in 1.5 ml of −80°C extraction solvent (40:40:20 acetonitrile-methanol-water) for cell quenching and metabolite extraction. Samples were then centrifuged (10,000 rpm, 4°C, 5 min), and 1 ml of cell-free supernatant was collected and stored at −80°C for metabolomic analysis. The time zero sample corresponded to the period directly before ^13^C-label addition. The ratio of ^13^C to ^12^C dissolved inorganic carbon remained constant during the course of the 2-h experimental period as determined by gas chromatography-mass spectrometry (GC-MS) analysis as described below.

### Formate batch experiments.

*N. moscoviensis* cells were harvested from the membrane bioreactor. The biomass was centrifuged at 8,000 × *g* for 15 min at 25°C and washed by resuspending the cells in fresh mineral NOB medium. This was repeated until no nitrite or nitrate was detectable via nitrite/nitrate test strips (Nitrite test strips MQuant; Merck) in the culture. Subsequently, the cells were transferred to sterile 120-ml serum bottles (triplicate bottles per time point, 6 time points total) containing 50 ml NOB mineral salts medium with 0.5 mM sodium formate and no nitrite but 0.187 mM NH_4_Cl as the nitrogen source. Bottles were crimp sealed with a rubber stopper to allow monitoring of the gas headspace and were incubated at 39°C in the dark. Following 24 h of acclimation, 1 mM ^13^C-labeled sodium formate (Cambridge Isotopes Laboratories, MA, USA) was added to all incubations. At each time point (before addition and after 15, 30, 60, 180, and 300 min), the isotopic composition of the gas headspace was measured using GC-MS (see below), and subsequently, all 3 bottles corresponding to a given time point were sacrificed for metabolomics analysis. Bottle contents were filtered immediately using a vacuum pump, and metabolites were extracted using −80°C extraction solvent (40:40:20 acetonitrile-methanol-water) as described above.

For growth experiments, *N. moscoviensis* cells from the membrane bioreactor were harvested and washed until no nitrite or nitrate was detectable via nitrite/nitrate test strips (Nitrite test strips MQuant; Merck) (see above). The cells were transferred to sterile 120-ml serum bottles with 50 ml NOB mineral medium containing 0.187 mM NH_4_Cl as the nitrogen source and a pH adjusted to 6.6, 7, or 7.7 with 1 M KHCO_3_ and were sealed with rubber stoppers. The experiment was performed in duplicates (per pH value and substrate), and 5 mM sodium nitrite or 5 mM sodium formate was added to the incubations as the sole energy source. Growth was monitored for 7 days using optical density measurements at a wavelength of 600 nm.

### Metabolomic analysis.

Samples were analyzed using a high-performance liquid chromatography (HPLC)-MS system consisting of a Vanquish UHPLC system (Thermo Scientific) coupled with electrospray ionization (ESI; negative polarity) to a hybrid quadrupole high-resolution mass spectrometer (Q Exactive Orbitrap; Thermo Scientific) operated in full scan mode for the detection of targeted compounds based on their accurate masses. Properties of full MS-selected ion monitoring (SIM) included a resolution of 140,000, automatic gain control (AGC) target of 1E6, maximum isolation time (IT) of 40 ms, and scan range from 70 to 1,000 *m/z*. LC separation was achieved using an ACQUITY UPLC BEH C_18_ column (2.1- by 100-mm column, 1.7-μm particle size, part no. 186002352, serial number 02623521115711; Waters). Solvent A was 97/3 water-methanol with 10 mM tributylamine (TBA) adjusted to pH 8.1 to 8.2 with 9 mM acetic acid. Solvent B was 100% methanol. Total run time was 25 min with the following gradient: 0 min, 5% B; 2.5 min, 5% B; 5 min, 20% B; 7.5 min, 20% B; 13 min, 55% B; 15.5 min, 95% B; 18.5 min, 95% B; 19 min, 5% B; and 25 min, 5% B. Flow rate was 200 μl min^−1^. The autosampler and column temperatures were 4°C and 25°C, respectively. Mass isotopomer distributions were corrected for natural abundance using the method of Su et al. (46), and ^13^C enrichment values were calculated using the formula $(1/N)\sum_{i=1}^{N}\mathrm{Mi}\times i,$ where *N* is the number of carbon atoms in the metabolite and *Mi* is the fractional abundance of the *i*th mass isotopomer.

### GC-MS analysis of dissolved inorganic carbon isotopic fractions.

Isotopic fractions of dissolved inorganic carbon in the liquid media were measured based on a modified headspace method (47). Three milliliters of liquid culture was collected from the bioreactor with a syringe and directly filtered through a 0.45-μm filter and 26-gauge needle into a 120-ml bottle containing 1 ml 6 M HCl (strong acid) crimp sealed with a rubber stopper. Prior to adding the liquid sample, bottles and HCl were flushed with either 100% N_2_ or Ar gas to void the headspace of background CO_2_. Samples were equilibrated with the acid in the bottles for at least 1 h at room temperature to drive all dissolved inorganic carbon into the gas phase. Fifty-microliter gas samples were then collected in a gas-tight syringe with a needle (Hamilton) from the bottle’s headspace and the isotopic fractions of ^12^CO_2_ and ^13^CO_2_ were determined using a gas chromatograph (Agilent 6890 equipped with 6 ft Porapak Q and molecular sieve columns) coupled to a mass spectrometer (GC-MS) (Agilent 5975C GC MSD; Agilent, Santa Clara, CA).

### Large-scale discovery proteomics of whole-cell and membrane fractions.

**(i) Whole-cell fraction sample preparation and proteolytic digestion.** Biomass was harvested by centrifugation, washed once with Tris-EDTA buffer, pH 7.7, and frozen using liquid N_2_. Cell pellets were stored at −80°C until further processing. For protein extraction, cell pellets (445 mg wet weight) were resuspended in 5 ml 50 mM triethylammonium bicarbonate (TEAB) buffer, 1% sodium deoxycholate, pH 8, and mixed 1:1 with B-PER reagent (Thermo Fisher Scientific, Waltham, MA, USA). Cells were lysed by two rounds of sonification (Sonifier B-12 with microtip; Branson Sonic Power Company, Danbury, CT, USA) at setting 6 for 30 s, intermitted by cooling on ice. Cell debris and unopened cells were separated from the sample by centrifugation (14,000 × *g*, room temperature [RT], 10 min). Proteins were precipitated by addition of 250 μl 100% (wt/vol) trichloroacetic acid (TCA) to 1 ml of sample and incubation for 20 min on ice. Proteins were collected by centrifugation (14,000 rpm, RT, 5 min) and washed twice with ice-cold acetone. Next, the protein pellet was resuspended in 400 μl 50 mM TEAB buffer aided by a heating step at 40°C for 1 h with intermittent vortexing. One hundred microliters of the protein extract was diluted 5 times with 6 M urea, 200 mM ammonium bicarbonate (ABC) buffer followed by vortexing and a heating step for 20 min at 45°C at 1,450 rpm and 25 min of sonification. Acetonitrile (ACN) was added to a final concentration of 50%, followed by another heating step. A 200-μl aliquot of the sample was reduced by the addition of dithiothreitol (DTT) to a final concentration of 2.3 mM followed by incubation at 37°C for 1 h. Next, the sample was alkylated by the addition of iodoacetamide (IAM) to a final concentration of 3.75 mM followed by incubation in the dark at room temperature for 30 min. The sample was diluted with 200 mM ABC buffer to a concentration of urea of <1 M. Lastly, a 150-μl aliquot of the protein sample was digested with 5 μg of sequencing-grade trypsin (Promega, Madison, WI, USA) overnight at 37°C.

**(ii) Membrane fraction sample preparation and proteolytic digestion.** Biomass was harvested by centrifugation, washed once with Tris-EDTA (TE) buffer, and frozen using liquid N_2_. Cell pellets were stored at −80°C until further processing. For protein extraction, cell pellets (1.95 g wet weight) were resuspended in 13 ml 50 mM TEAB buffer, 1% sodium deoxycholate, pH 8, and mixed 1:1 with B-PER reagent (Thermo Fisher Scientific). Cells were lysed by three passages through a French press at 138 MPa pressure. Cell debris and unopened cells were pelleted from the sample by centrifugation (5,000 × *g*, 4°C, 15 min). The membrane fraction was prepared using ultracentrifugation (45,000 rpm, 4°C, 1 h on an Optima-90 ultracentrifuge; Beckman-Coulter, Brea, CA, USA) and washed once in 200 mM ABC buffer, followed by another ultracentrifugation step to collect the membrane pellet. Finally, the membrane pellet was resuspended in 400 μl 200 mM ABC, 1% *N*-dodecyl β-d-maltoside (DDM), and membrane proteins were solubilized overnight in an orbital shaker at 4°C. The sample was clarified by centrifugation (20,000 × *g*, RT, 15 min). Urea was added to the sample to a final concentration of 6 M. The sample was reduced by the addition of DTT to a final concentration of 2.3 mM followed by incubation at 37°C for 1 h. Next, the sample was alkylated by the addition of IAM to a final concentration of 3.75 mM, followed by incubation in the dark at RT for 30 min. The sample was diluted to a concentration of 1 mg/ml with 200 mM ABC, 1% DDM, and 6 M urea. Five aliquots of 100 μg protein each were digested or doubly digested using 2 μg of each selected proteinase; all digestion steps were performed at 37°C. The following different proteinases and combinations were tested: (i) LysC (Pierce), 4 h; (ii) trypsin (Promega), 4 h; (iii) chymotrypsin (Pierce), overnight; (iv) LysC, 4 h followed by trypsin, overnight; and (v) trypsin, 4 h followed by chymotrypsin, overnight. In digests with trypsin and chymotrypsin, ACN was added to a final concentration of 20%, and the concentration of urea in the sample was diluted to ≤1 M by the addition of ABC buffer.

*(a) Solid phase extraction for membrane protein digest.* Proteolytic digests were desalted using an Oasis HLB 96-well plate (Waters, Milford, MA, USA) according to the manufacturer’s protocol. The purified peptide eluate was further dried using a speed-vacuum concentrator.

*(b) Large-scale shotgun proteomics.* All dried peptide fractions were resuspended in H_2_O containing 3% acetonitrile and 0.1% formic acid using mild vortexing. An aliquot of every sample corresponding to approximately 100 to 200 ng protein digest was analyzed in duplicates using a one-dimensional shotgun proteomics approach (48). Briefly, 1 μl of sample was injected to a nano-liquid chromatography system consisting of an EASY nano LC 1200, equipped with an Acclaim PepMap RSLC RP C_18_ separation column (50 μm by 150 mm, 2 μm, and 100 Å), and a QE plus Orbitrap mass spectrometer (Thermo). The flow rate was maintained at 300 nl/min over a linear gradient using H_2_O containing 0.1% formic acid as solvent A, and 80% acetonitrile in H_2_O and 0.1% formic acid as solvent B. The soluble protein extract fractions were analyzed using a gradient from 5% to 30% solvent B over 90 min, and finally to 75% B over 25 min. The soluble membrane protein fractions were analyzed using a shorter gradient from 4% to 30% B over 32.5 min followed by a second step to 65% B over 12.5 min, and data were acquired in total over 50 min. In either case, the Orbitrap was operated in data-dependent acquisition mode acquiring peptide signals from 350 to 1,400 *m/z* at 70,000 resolution, where the top 10 signals were isolated from a window of 2.0 *m/z* and fragmented using a normalized collision energy (NCE) of 30. The AGC target was set to 1E5, at a maximum IT of 54 ms and 17,500 resolution.

*(c) Database search and data processing.* Raw shotgun proteomics data from membrane proteins and soluble protein extract fractions were analyzed and combined using PEAKS Studio 8.5 (Bioinformatics Solutions Inc., Waterloo, Canada). Database search was performed allowing 20-ppm parent ion and 0.02-Da fragment mass error tolerance. Search conditions further considered 3 missed cleavages for the respective enzymes used, carbamidomethylation as fixed and methionine oxidation and N/Q deamidation as variable modifications. Peptide spectra were matched against a *N. moscoviensis* sequence database (UniProt TrEMBL, June 2018, taxon identifier [Tax ID] 42253), which was modified by filtering out duplicated entries. The database search included the GPM cRAP contaminant database (https://www.thegpm.org/crap/) and a decoy fusion for determining false-discovery rates. Peptide spectrum matches were filtered against a 1% false-discovery rate (FDR), and protein identifications with 2 or more unique peptides were considered significant hits. For the prefractionated whole-cell proteome, proteins were counted if they were significantly identified in at least one of the subfractions.

### Transmembrane helix predictions.

The combined transmembrane topology and signal peptide prediction tool Phobius (http://phobius.sbc.su.se [49]) was employed for the prediction of membrane proteins. Since this tool is optimized to distinguish between transmembrane helices and signal peptides, we defined all proteins with a prediction of one or more transmembrane helices (TMHs) as membrane proteins.

### Transcriptome analysis.

To relate the proteome data to the previously published transcriptome of *N. moscoviensis* under stable cultivation conditions grown on nitrite (13), we reanalyzed the transcriptome data (GEO series accession number GSE123406) using the *N. moscoviensis* genome annotation version CP011801.1 as a reference, from which the UniProt protein sequences were derived (5). The transcriptomic reanalysis was performed as described by Mundinger et al. (13). In short, quality-filtered raw reads from IonTorrent PGM sequencing (minimum quality score of 0.05, maximum sequencing length of 300 bp, allowing two ambiguous nucleotides) were mapped to the *N. moscoviensis* genome (NCBI accession number CP011801.1) using the mapping tool BBMap v35.92 (https://sourceforge.net/projects/bbmap/) and counted using featureCounts (50) with the parameters minid-0.95 with fracOverlap-0.9 for a minimum alignment identity of 95% over 90% of the read length and ambig=random to assign reads with multiple top-scoring mapping locations randomly to a single location.

Since the genome includes several duplicated genes, the 4,790 protein coding sequences (CDSs) correspond to only 4,733 UniProt entries for nonidentical proteins. To directly compare transcription to proteome data, the transcriptome reads of those identical genes were thus summed up.

Expression levels of CDSs were compared by ranking them from high to low based on their reads per kilobase per million reads (RPKM) values. Additionally, the log_2_ fold to median was calculated for genome-wide visualizations of the gene expression levels in a Circos plot (51).

### Data availability.

The transcriptomic data set reanalyzed here can be found under the NCBI Gene Expression Omnibus data set accession number GSE123406.

The mass spectrometry proteomics raw data have been deposited to the ProteomeXchange Consortium (http://proteomecentral.proteomexchange.org) via the PRIDE partner repository (52) with the data set identifier PXD019583.

10.1128/mSystems.00173-21.8 TABLE S3 Proteome coverages of different digestion strategies and cell fractions. The total numbers of proteins (detected with number of unique peptides of ≥2) are given followed by the relative coverage percentage in parentheses. Download Table S3, XLSX file, 0.01 MB. Copyright © 2021 Lawson et al. 2021 Lawson et al. https://creativecommons.org/licenses/by/4.0/ This content is distributed under the terms of the Creative Commons Attribution 4.0 International license.

## Supplementary Material

Reviewer comments
