# Supplementary figures and images for: Investigating the Chemolithoautotrophic and Formate Metabolism of Nitrospira moscoviensis by Constraint-Based Metabolic Modeling and 13C-Tracer Analysis
Source: mSystems. 2021 Aug 17;6(4):e00173-21. doi: 10.1128/mSystems.00173-21 (PMC8407350; doi:10.1128/mSystems.00173-21)

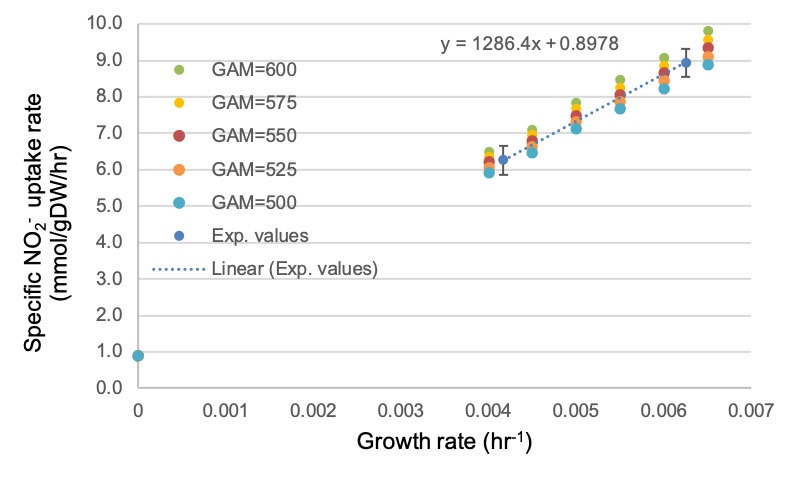

Supplement: FIG S1 [file msystems.00173-21-sf001.jpg]

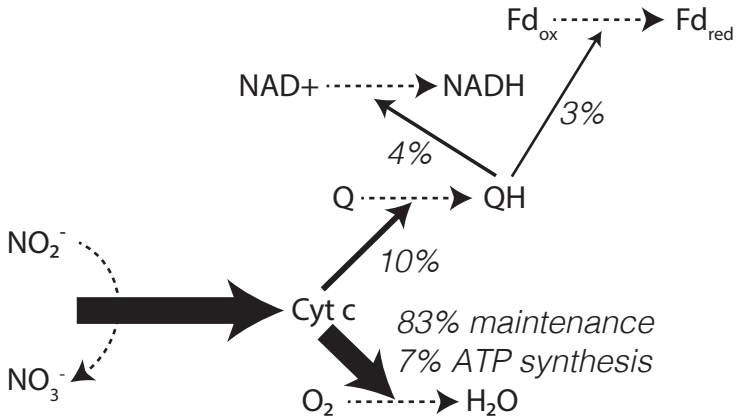

Supplement: FIG S2 [file msystems.00173-21-sf002.pdf]

**A**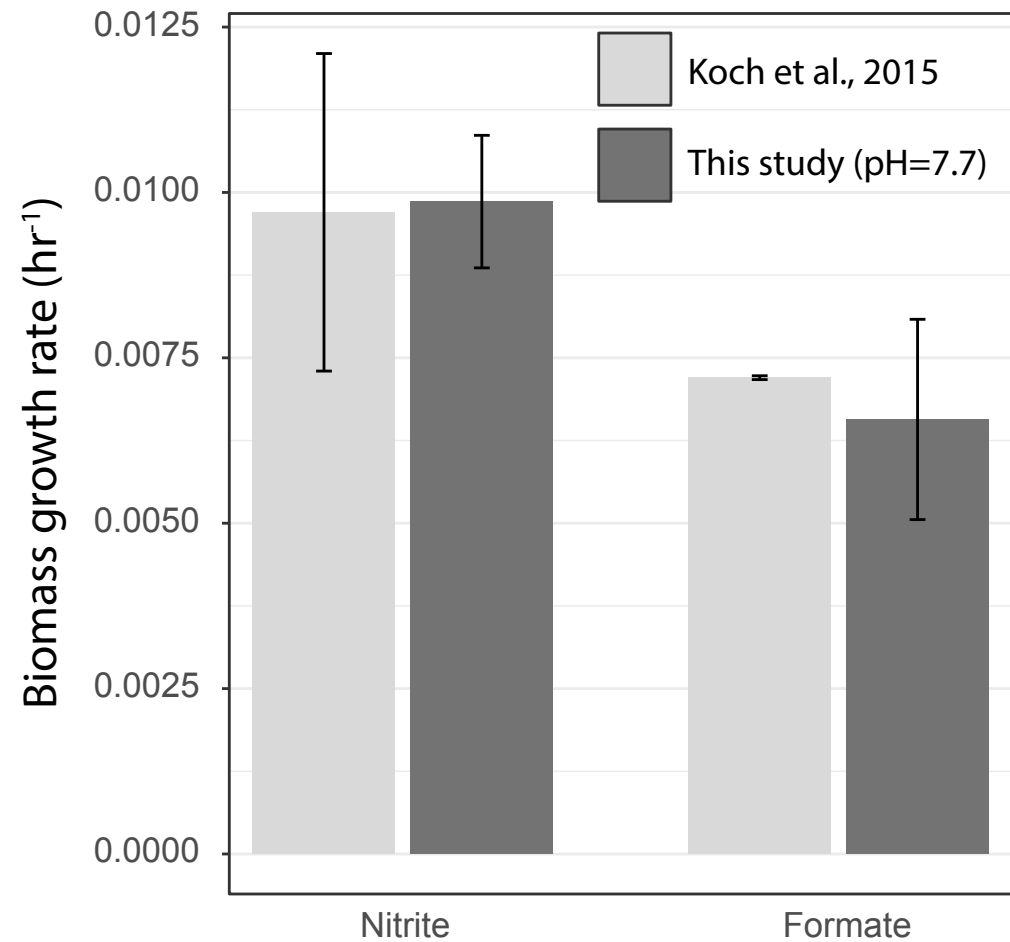**B**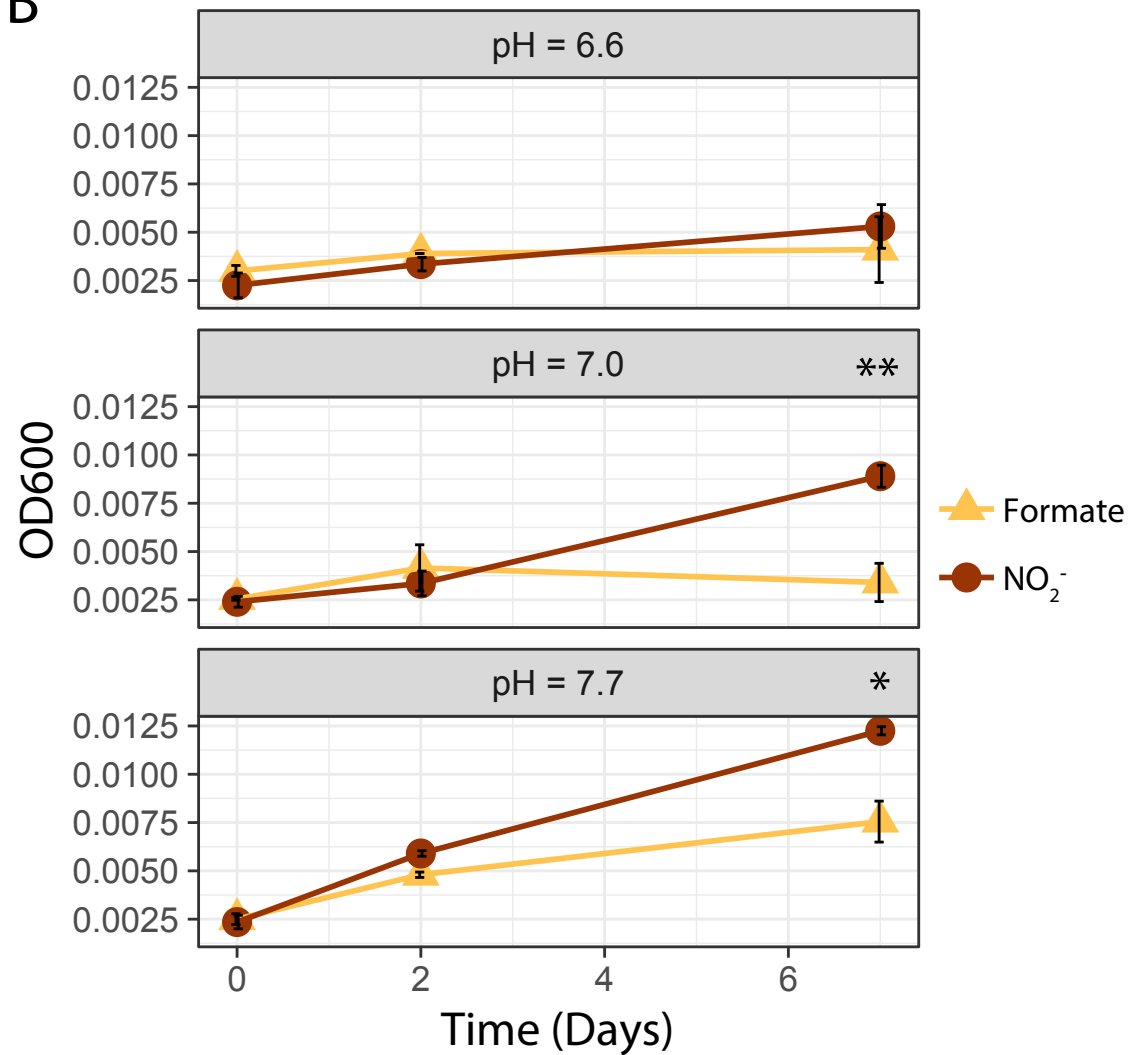

Supplement: FIG S4 [file msystems.00173-21-sf004.pdf]
