# Supplementary material for: Investigating the Chemolithoautotrophic and Formate Metabolism of Nitrospira moscoviensis by Constraint-Based Metabolic Modeling and 13C-Tracer Analysis
Source: mSystems. 2021 Aug 17;6(4):e00173-21. doi: 10.1128/mSystems.00173-21 (PMC8407350; doi:10.1128/mSystems.00173-21)
Supplement: TEXT S1 [file msystems.00173-21-t0001.pdf]

SUPPLEMENTARY INFORMATION

Investigating the chemolithoautotrophic and formate metabolism of *Nitrospira moscoviensis* by constraint-based metabolic modeling and ^{13}C-tracer analysis

Christopher E. Lawson[1,4,*,#], Aniela B. Mundinger[2,*], Hanna Koch[2], Tyler B. Jacobson[4], Coty A. Weathersby[1], Mike S. M. Jetten[2], Martin Pabst[3], Daniel Amador-Noguez[1,2], Daniel R. Noguera[1,4], Katherine McMahon[1,4,5], Sebastian Lücker[2,#]

[1]Department of Civil and Environmental Engineering, University of Wisconsin-Madison, Madison, WI, USA
[2]Department of Microbiology, Institute for Water and Wetland Research, Radboud University, Nijmegen, the Netherlands
[3]Department of Biotechnology, Delft University of Technology, Delft, The Netherlands
[4]DOE Great Lakes Bioenergy Research Center, University of Wisconsin-Madison, Madison, WI, USA
[5]Department of Bacteriology, University of Wisconsin-Madison, Madison, WI, USA
[*]These authors contributed equally to this work
[#]Corresponding authors: CEL (c.e.lawson.87@gmail.com), SL (s.luecker@science.ru.nl)

Supplementary Methods

Proteomic method validation

For an in-depth proteomic analysis of *N. moscoviensis* grown on nitrite, different cell fractions were analyzed and different protein digestion strategies were tested. A high coverage of membrane proteins is a prerequisite for analyzing complexes of the respiratory chain. However, membrane proteins are generally more difficult to detect in proteomics than soluble proteins, since their hydrophobic transmembrane helices contain generally less cleavage sides for digestion enzymes and make the protein less soluble. Therefore, enzymatic digests using three different digestion enzymes alone and in combinations were tested.

The coverages of the membrane proteomes ranged from 8.5 – 13.4%. While the majority of proteins were detected in all five different digests, there were uniquely detected proteins found with each of the different digestion protocols (Supplement Figure S3). The sequential protein digestion using LysC and Trypsin yielded to the highest coverage of the membrane proteome (Supplementary Table 1). The higher coverage was likely caused by the high tolerance of LysC to urea, allowing strong denaturing conditions during the first part of the digest, in combination with the advantage of a double digest, leading to fewer missed cleavage sides.

The proteomics approach using the whole cell fraction combined with a prefractionation of the peptides proved to be more powerful than any targeted analysis of only the membrane fraction. The whole cell proteome analysis resulted in the detection of 53.2 % of the 4733 of the non-identical proteins encoded in the *N. moscoviensis* genome including 39.2 % of 878 non-identical membrane proteins. A comparison of the relative protein coverage per transmembrane helices (TMH) demonstrated that only the whole cell proteome and the LysC/Trypsin membrane proteome detected proteins with 16 or 17 TMH (Supplementary Figure S3). Apart from this there was no clear trend between coverage and TMH, which would indicate a bias against hydrophobic membrane proteins.
