# Supplementary material for: Investigating the Chemolithoautotrophic and Formate Metabolism of Nitrospira moscoviensis by Constraint-Based Metabolic Modeling and 13C-Tracer Analysis
Source: mSystems. 2021 Aug 17;6(4):e00173-21. doi: 10.1128/mSystems.00173-21 (PMC8407350; doi:10.1128/mSystems.00173-21)
Supplement: FIG S3 [file msystems.00173-21-sf003.pdf]

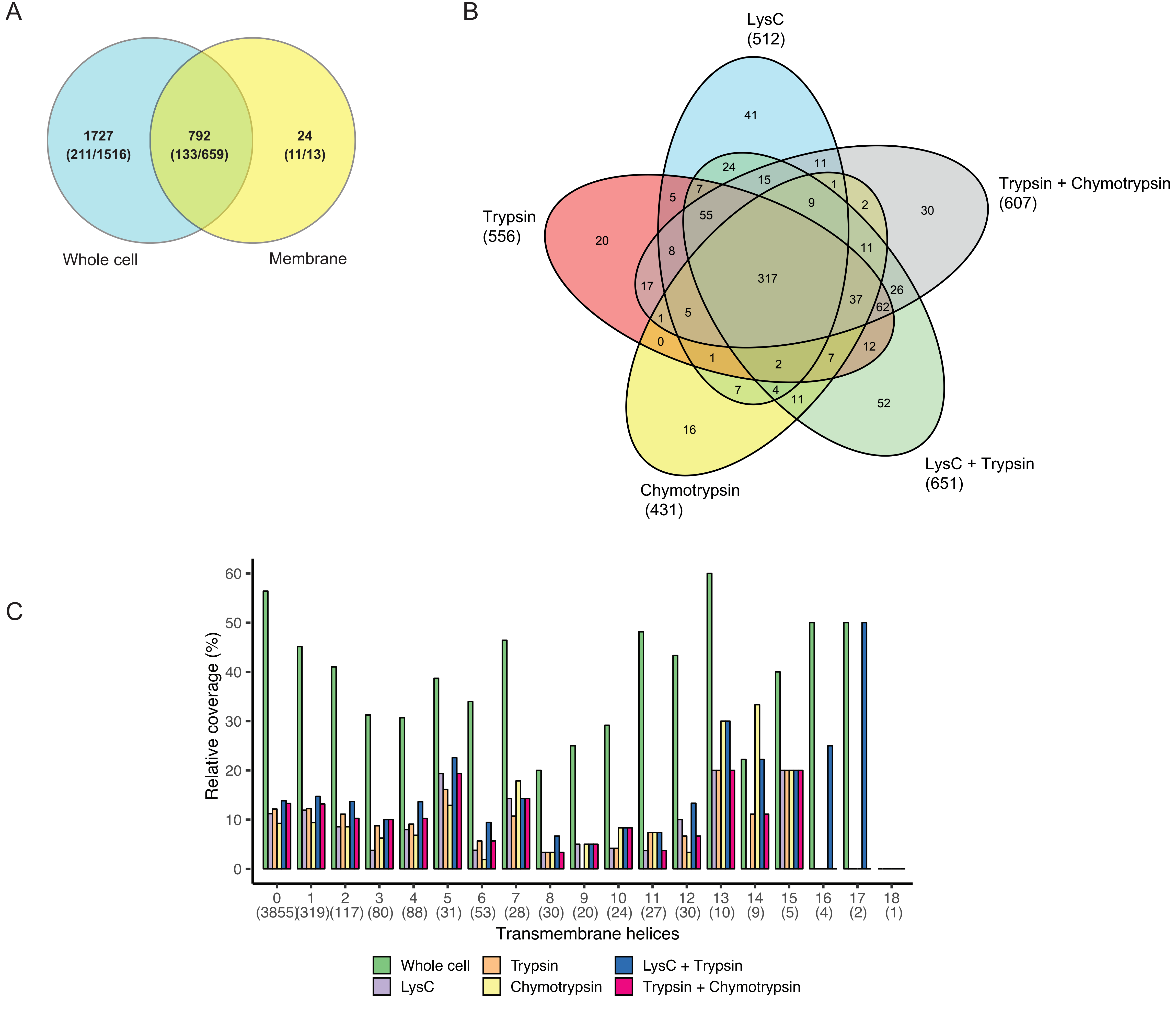
A
1727
(211/1516)
792
(133/659)
24
(11/13)
Whole cell
Membrane
B
LysC
(512)
Trypsin
(556)
Trypsin + Chymotrypsin
(607)
LysC + Trypsin
(651)
Chymotrypsin
(431)
41
24
11
15
1
5
7
9
2
55
30
20
8
11
317
17
26
37
62
1
5
0
12
1
2
7
7
4
11
52
16
C
Relative coverage (%)
0
10
20
30
40
50
60
Transmembrane helices
0 (3855)
1 (319)
2 (117)
3 (80)
4 (88)
5 (31)
6 (53)
7 (28)
8 (30)
9 (20)
10 (24)
11 (27)
12 (30)
13 (10)
14 (9)
15 (5)
16 (4)
17 (2)
18 (1)
Whole cell
LysC
Trypsin
Chymotrypsin
LysC + Trypsin
Trypsin + Chymotrypsin
