# Supplementary material for: Investigating the Chemolithoautotrophic and Formate Metabolism of Nitrospira moscoviensis by Constraint-Based Metabolic Modeling and 13C-Tracer Analysis
Source: mSystems. 2021 Aug 17;6(4):e00173-21. doi: 10.1128/mSystems.00173-21 (PMC8407350; doi:10.1128/mSystems.00173-21)
Supplement: Reviewer comments [file reviewer-comments.pdf]

AMERICAN SOCIETY FOR MICROBIOLOGY | mSystems®

Investigating the chemolithoautotrophic and formate metabolism of *Nitrospira moscoviensis* by constraint-based metabolic modeling and ^{13}C-tracer analysis

Christopher Lawson, Aniela Mundinger, Hanna Koch, Tyler Jacobson, Coty Weathersby, Mike Jetten, Martin Pabst, Daniel Amador-Noguez, Daniel Noguera, Katherine McMahon, and Sebastian Lücker

Corresponding Author(s): Christopher Lawson, University of Wisconsin-Madison

Review Timeline:

Submission Date:	February 16, 2021
Editorial Decision:	April 1, 2021
Revision Received:	July 6, 2021
Accepted:	July 21, 2021

Editor: Steven Hallam

Reviewer(s): Disclosure of reviewer identity is with reference to reviewer comments included in decision letter(s). The following individuals involved in review of your submission have agreed to reveal their identity: Brett L Mellbye (Reviewer #1)

Transaction Report:

(Note: With the exception of the correction of typographical or spelling errors that could be a source of ambiguity, letters and reports are not edited. The original formatting of letters and referee reports may not be reflected in this compilation.)

DOI: https://doi.org/10.1128/mSystems.00173-21

April 1, 2021

Dr. Christopher E Lawson
University of Wisconsin-Madison
Cuvil abd Environmental Engineering
Madison

Re: mSystems00173-21 (Investigating the chemolithoautotrophic and formate metabolism of *Nitrospira moscoviensis* by constraint-based metabolic modeling and ^{13}C-tracer analysis)

Dear Dr. Christopher E Lawson:

Thank you for your submission to the journal. Overall, the external reviewers provided positive responses and the work is almost ready for publication. Please look carefully over the reviewer comments and determine the extend to which additional work is warranted. In addition, please develop a "Data Availability" section at the end of the Materials and Methods section to guide other researchers to the information used in your analysis in support of reproducible research and open science practices.

Below you will find the comments of the reviewers.

To submit your modified manuscript, log onto the eJP submission site at https://msystems.msubmit.net/cgi-bin/main.plex. If you cannot remember your password, click the "Can't remember your password?" link and follow the instructions on the screen. Go to Author Tasks and click the appropriate manuscript title to begin the resubmission process. The information that you entered when you first submitted the paper will be displayed. Please update the information as necessary. Provide (1) point-by-point responses to the issues raised by the reviewers as file type "Response to Reviewers," not in your cover letter, and (2) a PDF file that indicates the changes from the original submission (by highlighting or underlining the changes) as file type "Marked Up Manuscript - For Review Only."

Due to the SARS-CoV-2 pandemic, our typical 60 day deadline for revisions will not be applied. I hope that you will be able to submit a revised manuscript soon, but want to reassure you that the journal will be flexible in terms of timing, particularly if experimental revisions are needed. When you are ready to resubmit, please know that our staff and Editors are working remotely and handling submissions without delay. If you do not wish to modify the manuscript and prefer to submit it to another journal, please notify me of your decision immediately so that the manuscript may be formally withdrawn from consideration by mSystems.

If your manuscript is accepted for publication, you will be contacted separately about payment when the proofs are issued; please follow the instructions in that e-mail. Arrangements for payment must be made before your article is published. For a complete list of **Publication Fees**, including supplemental material costs, please visit our website.

Corresponding authors may join or renew ASM membership to obtain discounts on publication fees. Need to upgrade your membership level? Please contact Customer Service at Service@asmusa.org.

Thank you for submitting your paper to mSystems.

Sincerely,

Steven Hallam

Editor, mSystems

Journals Department
American Society for Microbiology
1752 N St., NW
Washington, DC 20036
E-mail: peerreview@asmusa.org
Phone: 1-202-942-9338

Reviewer comments:

Reviewer #1 (Comments for the Author):

Please see attached file.

Reviewer #2 (Comments for the Author):

Lawson and coworkers present a study using comparative proteomics, metabolic flux modeling, and "pulse chase" metabolic labeling experiments to investigate chemoautotrophic vs. (putatively) heterotrophic growth in a model nitrite-oxidizing bacterium. This is an interesting paper using methods that have been applied frequently to heterotrophic microbes, but rarely (ever?) to chemolithoautotrophs. Interesting findings from the paper include insight into the mechanism of growth on formate and potential for "substrate channeling" to increase enzymatic efficiency. However, overall I would say the strength of the paper relies in demonstrating the feasibility of the approach for what are normally considered very fastidious organisms and the useful cell composition measurements.

General comment - I don't understand the authors' usage of the terms "constraint-based analysis" versus "flux balance analysis." I'm much more familiar with the later term. Can this be clarified?

It would be clearer if the model were just referred to as "the model" throughout the manuscript. The way it is referred to by name in some places causes confusion about whether it is a microbial strain. (For example Line 131)

Specific comments:

line 65 - I don't know that I would say the CBB cycle is "common." All Nitrospina also use the rTCA cycle.

line 70 - Nitrospira marina also uses formate (Bayer et al. 2020, ISMEJ)

line 92 - I'd delete "canonical" here

line 109- I don't understand what "reactions carrying flux" means.

line 134 - Can you elaborate here on exactly what is meant by "maximization of biomass growth"? That cells are trying to maximize dry weight per cell?

The Discussion would benefit from some general comparisons of the proteome (e.g. fraction detected) with other NOB (the aforementioned Bayer et al. paper for example) or even AOB.

lines 301 - I'm not sure what is meant by this statement, that the model "quantitatively predicts chemolithoautotrophic growth on nitrite."? Wasn't that known? Do any of the model outputs allow for a prediction of the amount of C fixed per N oxidized?

Figure 3 - Oxygen stoichiometry for NO2:O2 should be 2:1. What is causing the discrepancy?

Figure 6 - Given that the model predictions didn't play out for the formate utilization, this figure is a bit misleading. Is there a way to incorporate "predicted" vs. "actual" in some way?

Table 1 - Clarify in the caption that these are percentages. Also some typos in this table.

This study demonstrates the utility of genome-scale, constraints-based modeling to investigate metabolism in *Nitrospira moscoviensis* as well as other systems. The authors reconstructed a genome-scale metabolic model and used a constraints-based analysis to investigate growth on formate, nitrite, and formate+nitrite. The modeling work suggested drastic differences in growth rate and biomass accumulation depending on how formate and CO2 were incorporated and led to three questions: 1. How does *Nitrospira moscoviensis* fix CO2? 2. How is formate incorporated during mixotrophic growth? 3. Can *N. moscoviensis* grow faster using formate as a sole energy source?

By using the power of proteomics and 13C tracer metabolomics analyses in tandem along with growth experiments, the authors were able to address these questions and demonstrate the value of model-driven hypothesis investigation. Proteomic analysis provided evidence of which genes were important in reverse electron flow, 13C bicarbonate-tracer metabolomics provided evidence of CO2 fixation via the rTCA cycle, and 13C formate-tracer metabolomics and growth experiments suggests formate C is incorporated via CO2 fixation and growth rate on formate is slower than nitrite. An additional finding from metabolomics was potential compartmentalization or substrate channeling of some metabolites. Overall, this study substantially expands our knowledge of *Nitrospira moscoviensis* physiology and provides a wealth of data to generate future research questions.

General comments:

Overall, this manuscript was a pleasure to read and did a great job communicating a substantial amount of data and a complex topic. My biggest suggestion to improve the manuscript is to confirm growth experiments with more replication and provide a better comparison and explanation of model predictions and actual growth experiment data.

1. All of the growth experiments are N = 2. Usually at least 3 replicates are needed to confirm a statistically significant difference between treatments. Can the authors confirm that there was statistically significant difference between formate and nitrite treatments?
2. Since the model had such drastically different predictions of growth rate, biomass, etc. depending on growth on formate or nitrite, I would really like to see model predictions graphed alongside the growth experiments or a comparison of predicted vs. actual growth parameters.
3. Have the authors experimented with modifying the metabolic constraints to better predict growth on formate? Formate being “toxic” seems unlikely. A more likely hypothesis is that there is a metabolic constraint that prevents efficient growth on formate as a sole energy source.
4. Did the authors measure growth rate and biomass growing on both formate and nitrite? What are potential metabolic differences between growth on only nitrite, nitrite + formate, or only formate? The model provides the framework to investigate these questions by making predictions and modifying constraints. Please address this in the text.

Minor comments:

Line 539: Please define DDM in the text.

Line 569: I think you mean data-dependent acquisition mode here.

line 592: Please define TMH in the text.

References: Please check to make sure species names are in italics.

Figure 6: It would be helpful if this figure also included the actual experimental growth rate measured in wet lab experiments.

Tables 1 and 2: These tables need to be re-formatted before publication.

Reviewer #1
This study demonstrates the utility of genome-scale, constraints-based modeling to investigate metabolism in Nitrospira moscoviensis as well as other systems. The authors reconstructed a genome- scale metabolic model and used a constraints-based analysis to investigate growth on formate, nitrite, and formate+nitrite. The modeling work suggested drastic differences in growth rate and biomass accumulation depending on how formate and CO2 were incorporated and led to three questions: 1. How does Nitrospira moscoviensis fix CO2? 2. How is formate incorporated during mixotrophic growth? 3. Can N. moscoviensis grow faster using formate as a sole energy source?
By using the power of proteomics and 13C tracer metabolomics analyses in tandem along with growth experiments, the authors were able to address these questions and demonstrate the value of model- driven hypothesis investigation. Proteomic analysis provided evidence of which genes were important in reverse electron flow, 13C bicarbonate-tracer metabolomics provided evidence of CO2 fixation via the rTCA cycle, and 13C formate-tracer metabolomics and growth experiments suggests formate C is incorporated via CO2 fixation and growth rate on formate is slower than nitrite. An additional finding from metabolomics was potential compartmentalization or substrate channeling of some metabolites. Overall, this study substantially expands our knowledge of Nitrospira moscoviensis physiology and provides a wealth of data to generate future research questions.

General comments:
Overall, this manuscript was a pleasure to read and did a great job communicating a substantial amount of data and a complex topic. My biggest suggestion to improve the manuscript is to confirm growth experiments with more replication and provide a better comparison and explanation of model predictions and actual growth experiment data.

We thank the reviewer for their positive comments on the manuscript and have addressed each point raised by the reviewer below.

1. All of the growth experiments are N = 2. Usually at least 3 replicates are needed to confirm a statistically significant difference between treatments. Can the authors confirm that there was statistically significant difference between formate and nitrite treatments?

We have re-plotted the growth data with error bars representing 1 standard deviation and conducted a student t-test between treatments, which indicates that the differences between growth on formate and nitrite were statistically significant, even with 2 replicates. The new figure has been added to the manuscript (now FigS4), along with an updated figure legend denoting statistical significance.

2. Since the model had such drastically different predictions of growth rate, biomass, etc. depending on growth on formate or nitrite, I would really like to see model predictions graphed alongside the growth experiments or a comparison of predicted vs. actual growth parameters.

We prefer to avoid providing a direct comparison of the predicted and actual formate growth data because the model was calibrated on chemostat data, whereas the growth on formate data was derived from batch experiments (both in Koch 2015 and our study). When we compared observed growth on nitrite in batch vs chemostat cultures (substrate limited) we saw discrepancies in substrate uptake rates. Since the model was calibrated on the chemostat data, this leads to an underestimation of the predicted growth rate on nitrite compared to the batch culture data, which is why we avoided direct comparisons. We tried but were unable to collect chemostat data for formate growth because the reactor culture on formate was unstable, making it not possible to collect detailed kinetic parameters. Nonetheless, the model was still capable of predicting the expected higher growth rate on formate correctly, which is the overall point and is contradictory the measured data.

However, we still agree that it is helpful to visualize the observed slower growth on formate vs nitrite in a similar format to the presented model predictions and have therefore provided this in Figure S4.

3. Have the authors experimented with modifying the metabolic constraints to better predict growth on formate? Formate being "toxic" seems unlikely. A more likely hypothesis is that there is a metabolic constraint that prevents efficient growth on formate as a sole energy source.

We have proposed a model-driven hypothesis to explain why growth on formate is less than growth on nitrite, based on complex I being constrained or limited in the oxidative direction (i.e., oxidation of NADH [NADH + 4.5 H+ + Q => NAD + 3.5 H+[e] + QH2]). Under such a scenario, electrons from formate oxidation (i.e., NADH) would be slower to enter the electron transport chain resulting in an electron imbalance that would have to be alleviated by the secretion of a reduced product. From our analysis, we predict that these electrons could be balanced via central carbon reactions; for example, the reduction of oxaloacetate to malate mediated by malate dehydrogenase (NAD + Malate <= NADH + Oxaloacetate + H+). This would divert fixed carbon from biomass into the production and secretion of a central metabolite, resulting in reduced growth. This model generated hypothesis has been added to the results and discussion section of the manuscript (Page 10-11, lines 290-306; Page 12-13, lines 360-367).

4. Did the authors measure growth rate and biomass growing on both formate and nitrite? What are potential metabolic differences between growth on only nitrite, nitrite + formate, or only formate? The model provides the framework to investigate these questions by making predictions and modifying constraints. Please address this in the text.

Fig 6 presents modeling scenarios that compare predicted growth on nitrite, formate, and nitrite+formate. However, as discussed in the manuscript, the predicted growth on formate did not agree with the measured growth on formate. While we did not measure growth on both formate and nitrite, Koch et al., 2015 could show that formate and nitrite were consumed by N. moscoviensis simultaneously. However, nitrite oxidation rates strongly decreased in the presence of formate. Based on this, we posit that the mechanism described above in point #3

would also constrain electron flow through the ETC from formate, resulting in reduced growth due to electron imbalances.

Minor comments:
Line 539: Please define DDM in the text.

We have defined N-dodecyl β-D-maltoside (DDM) in the manuscript.

Line 569: I think you mean data-dependent acquisition mode here. line 592: Please define TMH in the text.

We have corrected the spelling of data-dependent acquisition mode and defined transmembrane helix (TMH)

References: Please check to make sure species names are in italics.

We have corrected the italics of species names in the references.

Figure 6: It would be helpful if this figure also included the actual experimental growth rate measured in wet lab experiments.

Please see response to comment #2

Tables 1 and 2: These tables need to be re-formatted before publication.

We will reformat Tables 1 and 2 based on recommendations from the publishing team.

Reviewer #2 (Comments for the Author):

Lawson and coworkers present a study using comparative proteomics, metabolic flux modeling, and "pulse chase" metabolic labeling experiments to investigate chemoautotrophic vs. (putatively) heterotrophic growth in a model nitrite-oxidizing bacterium. This is an interesting paper using methods that have been applied frequently to heterotrophic microbes, but rarely (ever?) to chemolithoautotrophs. Interesting findings from the paper include insight into the mechanism of growth on formate and potential for "substrate channeling" to increase enzymatic efficiency. However, overall I would say the strength of the paper relies in demonstrating the feasibility of the approach for what are normally considered very fastidious organisms and the useful cell composition measurements.

We thank the reviewer for their positive feedback on the manuscript.

General comment - I don't understand the authors' usage of the terms "constraint-based analysis" versus "flux balance analysis." I'm much more familiar with the later term. Can this be clarified?

Flux balance analysis and constraint-based analysis are often used interchangeably in the modeling community, however, for clarity we have changed constraint-based analysis to flux balance analysis in the paper (pg 1, line 23).

It would be clearer if the model were just referred to as "the model" throughout the manuscript. The way it is referred to by name in some places causes confusion about whether it is a microbial strain. (For example Line 131)

We have changed “iNmo686” to “the model” throughout the manuscript for clarity.

Specific comments:

line 65 - I don't know that I would say the CBB cycle is "common." All Nitrospina also use the rTCA cycle.

We have changed “common” to “used by some NOB”

line 70 - Nitrospira marina also uses formate (Bayer et al. 2020, ISMEJ)

We have included this reference in introduction L70 and discussion (pg 12, line 364-365).

line 92 - I'd delete "canonical" here

We have deleted the word canonical.

line 109- I don't understand what "reactions carrying flux" means.

We have changed “reactions carrying flux” to “reactions with non-zero flux values”

line 134 - Can you elaborate here on exactly what is meant by "maximization of biomass growth"? That cells are trying to maximize dry weight per cell?

We have updated this sentence to state that maximization of biomass growth “rate” is the objective

The Discussion would benefit from some general comparisons of the proteome (e.g. fraction detected) with other NOB (the aforementioned Bayer et al. paper for example) or even AOB.

We agree with the reviewer and included the findings of Bayer et al. in the discussion (pg 12, line 364-365).

lines 301 - I'm not sure what is meant by this statement, that the model "quantitatively predicts chemolithoautotrophic growth on nitrite."? Wasn't that known? Do any of the model outputs allow for a prediction of the amount of C fixed per N oxidized?

Yes, we have added the amount of C fixed per N oxidized (Pg 7, line 191-192)

Figure 3 - Oxygen stoichiometry for NO2:O2 should be 2:1. What is causing the discrepancy?

Some nitrite is assimilated for growth, increasing the NO2:O2 ratio slightly above 2:1

Figure 6 - Given that the model predictions didn't play out for the formate utilization, this figure is a bit misleading. Is there a way to incorporate "predicted" vs. "actual" in some way?

As discussed above for reviewer #1 comment #2- we prefer to avoid providing a direct comparison of the predicted and actual formate growth data because the model was calibrated on chemostat data, whereas the growth on formate data was derived from batch experiments (both in Koch 2015 and our study). When we compared observed growth on nitrite in batch vs chemostat cultures (substrate limited) we saw discrepancies in substrate uptake rates. Since the model was calibrated on the chemostat data, this leads to an underestimation of the predicted growth rate on nitrite compared to the batch culture data, which is why we avoided direct comparisons. We tried but were unable to collect chemostat data for formate growth because the reactor culture on formate was unstable, making it not possible to collect detailed kinetic parameters. Nonetheless, the model was still capable of predicting the expected higher growth rate on formate correctly, which is the overall point and is contradictory the measured data.

However, we still agree that it is helpful to visualize the observed slower growth on formate vs nitrite in a similar format to the presented model predictions and have therefore provided this in Figure S4.

Table 1 - Clarify in the caption that these are percentages. Also some typos in this table.

Thanks, we have clarified that the reported values are percentages and corrected the typos.

July 21, 2021

Dr. Christopher E Lawson
University of Wisconsin-Madison
Cuvil abd Environmental Engineering
Madison

Re: mSystems00173-21R1 (Investigating the chemolithoautotrophic and formate metabolism of *Nitrospira moscoviensis* by constraint-based metabolic modeling and ^{13}C-tracer analysis)

Dear Dr. Christopher E Lawson:

Your manuscript has been accepted, and I am forwarding it to the ASM Journals Department for publication. For your reference, ASM Journals' address is given below. Before it can be scheduled for publication, your manuscript will be checked by the mSystems senior production editor, Ellie Ghatineh, to make sure that all elements meet the technical requirements for publication. She will contact you if anything needs to be revised before copyediting and production can begin. Otherwise, you will be notified when your proofs are ready to be viewed.

As an open-access publication, mSystems receives no financial support from paid subscriptions and depends on authors' prompt payment of publication fees as soon as their articles are accepted. =

Publication Fees:
You will be contacted separately about payment when the proofs are issued; please follow the instructions in that e-mail. Arrangements for payment must be made before your article is published. For a complete list of **Publication Fees**, including supplemental material costs, please visit our website.

Corresponding authors may join or renew ASM membership to obtain discounts on publication fees. Need to upgrade your membership level? Please contact Customer Service at Service@asmusa.org.

For mSystems research articles, you are welcome to submit a short author video for your recently accepted paper. Videos are normally 1 minute long and are a great opportunity for junior authors to get greater exposure. Importantly, this video will not hold up the publication of your paper, and you can submit it at any time.

Details of the video are:

· Minimum resolution of 1280 x 720
· .mov or .mp4. video format
· Provide video in the highest quality possible, but do not exceed 1080p
· Provide a still/profile picture that is 640 (w) x 720 (h) max
· Provide the script that was used

We recognize that the video files can become quite large, and so to avoid quality loss ASM suggests sending the video file via https://www.wetransfer.com/. When you have a final version of the video and the still ready to share, please send it to Ellie Ghatineh at eghatineh@asmusa.org.

Thank you for submitting your paper to mSystems.

Sincerely,

Steven Hallam
Editor, mSystems

Journals Department
American Society for Microbiology
1752 N St., NW
Washington, DC 20036
E-mail: peerreview@asmusa.org
Phone: 1-202-942-9338

Fig. S4: Accept
Table S2: Accept
Fig. S3: Accept
Table S3: Accept
Fig. S2: Accept
Table S1: Accept
Fig. S1: Accept
Supplemental Data 1: Accept
Supplemental Material: Accept
Supplemental Data 2: Accept
